# Dysnumeria in Sign Language: Impaired Construction of the Decimal Structure in Reading Multidigit Numbers in a Deaf ISL Signer

**DOI:** 10.3389/fpsyg.2021.649109

**Published:** 2021-06-29

**Authors:** Naama Friedmann, Neta Haluts, Doron Levy

**Affiliations:** Language and Brain Lab, Tel Aviv University, Tel Aviv, Israel

**Keywords:** number impairment, sign language, number reading, dyscalculia, deaf, number reading model, reading, dysnumeria

## Abstract

We report on the first in-depth analysis of a specific type of dysnumeria, number-reading deficit, in sign language. The participant, Nomi, is a 45-year-old signer of Israeli Sign Language (ISL). In reading multidigit numbers (reading-then-signing written numbers, the counterpart of reading aloud in spoken language), Nomi made mainly decimal, number-structure errors– reading the correct digits in an incorrect (smaller) decimal class, mainly in longer numbers of 5–6-digits. A unique property of ISL allowed us to rule out the numeric-visual analysis as the source of Nomi's dysnumeria: In ISL, when the multidigit number signifies the number of objects, it is signed with a decimal structure, which is marked morphologically (e.g., 84 → Eight-Tens Four); but a parallel system exists (e.g., for height, age, bus numbers), in which multidigit numbers are signed non-decimally, as a sequence of number-signs (e.g., 84 → Eight, Four). When Nomi read and signed the exact same numbers, but this time non-decimally, she performed significantly better. Additional tests supported the conclusion that her early numeric-visual abilities are intact: she showed flawless detection of differences in length, digit-order, or identity in same-different tasks. Her decimal errors did not result from a number-structure deficit in the phonological-sign output either (no decimal errors in repeating the same numbers, nor in signing multidigit numbers written as Hebrew words). Nomi had similar errors of conversion to the decimal structure in number comprehension (number-size comparison tasks), suggesting that her deficit is in a component shared by reading and comprehension. We also compared Nomi's number reading to her reading and signing of 406 Hebrew words. Nomi's word reading was in the high range of the normal performance of hearing controls and of deaf signers and significantly better than her multidigit number reading, demonstrating a dissociation between number reading, which was impaired, and word reading, which was spared. These results point to a specific type of dysnumeria in the number-frame generation for written multidigit numbers, whereby the conversion from written multidigit numbers to the abstract decimal structure is impaired, affecting both reading and comprehension. The results support abstract, non-verbal decimal structure generation that is shared by reading and comprehension, and also suggest the existence of a non-decimal number-reading route.

## 1. Introduction

Number reading, just like word reading, is a complex, multi-staged process (McCloskey et al., 1985, 1986, 1990; Cohen et al., 1997; Dehaene et al., 2003; Dotan and Friedmann, 2018), which is essential in our everyday life (Nuerk et al., 2015). A deficit in each of the components of this number-reading process gives rise to a different type of dysnumeria, which manifests itself in different types of errors and in different patterns of performance in various number tasks (McCloskey et al., 1985, 1986, 1990; Temple, 1989; Noel and Seron, 1993; Cipolotti and Butterworth, 1995; Cipolotti et al., 1995; Cohen et al., 1997; Basso and Beschin, 2000; Deloche and Willmes, 2000; Delazer and Bartha, 2001; Dehaene et al., 2003; Cappelletti et al., 2005; Friedmann et al., 2010; Starrfelt et al., 2010; Starrfelt and Behrmann, 2011; Moura et al., 2013; Dotan and Friedmann, 2015, 2018). Until now, dysnumerias have been reported for spoken languages. In this paper we report on the first in-depth analysis of a specific dysnumeria in sign language, in Nomi, a 45-year-old signer of Israeli Sign Language (ISL).

Previous studies reported that compared to hearing individuals, deaf individuals have difficulties with numbers, mostly in arithmetic and mathematics (Wollman, 1964; Austin, 1975; Wood et al., 1984; Titus, 1995; Frostad, 1996; Nunes and Moreno, 1998; Traxler, 2000; Davis and Kelly, 2003; Bull et al., 2011; Gottardis et al., 2011). However, these studies referred to the deaf population as a whole, without examining signers specifically, and they referred to perfomance in general mathematics tests, without assessing number-reading. Additionally, these reports are mainly obtained from general math tests administered in English, and these have been associated with difficulties in the spoken language (cf. Kelly and Gaustad, 2007, where the performance in mathematics tests was found to correlate with the performance in reading and morphology in English). Other studies have focused on specific properties of sign languages and their effects on number processing, e.g., how the sub-base 5 of the numeral system in the German sign language, Deutsche Gebärdensprache (DGS) affects parity retrieval (Iversen et al., 2006), and how DGS properties affect the response times on parity judgmenets with the left or right hand (MARC effect, Iversen et al., 2004), but have not related to specific difficulties in number processing. The studies that did assess number-reading difficulties in deaf individuals have not tested signing of multidigit numbers (Genovese et al., 2005; Korvorst et al., 2007; Domahs et al., 2010, 2012; Palma et al., 2010). As a consequence, no study has analyzed the pattern of errors made by deaf signers in reading multidigit numbers, and there have been no reports of selective impairments in number-reading in sign language. However, as we show below, testing dysnumeria in sign language offers interesting insights to dysnumeria and to the number-reading model.

### 1.1. The Number Reading Process

**Figure 1:**
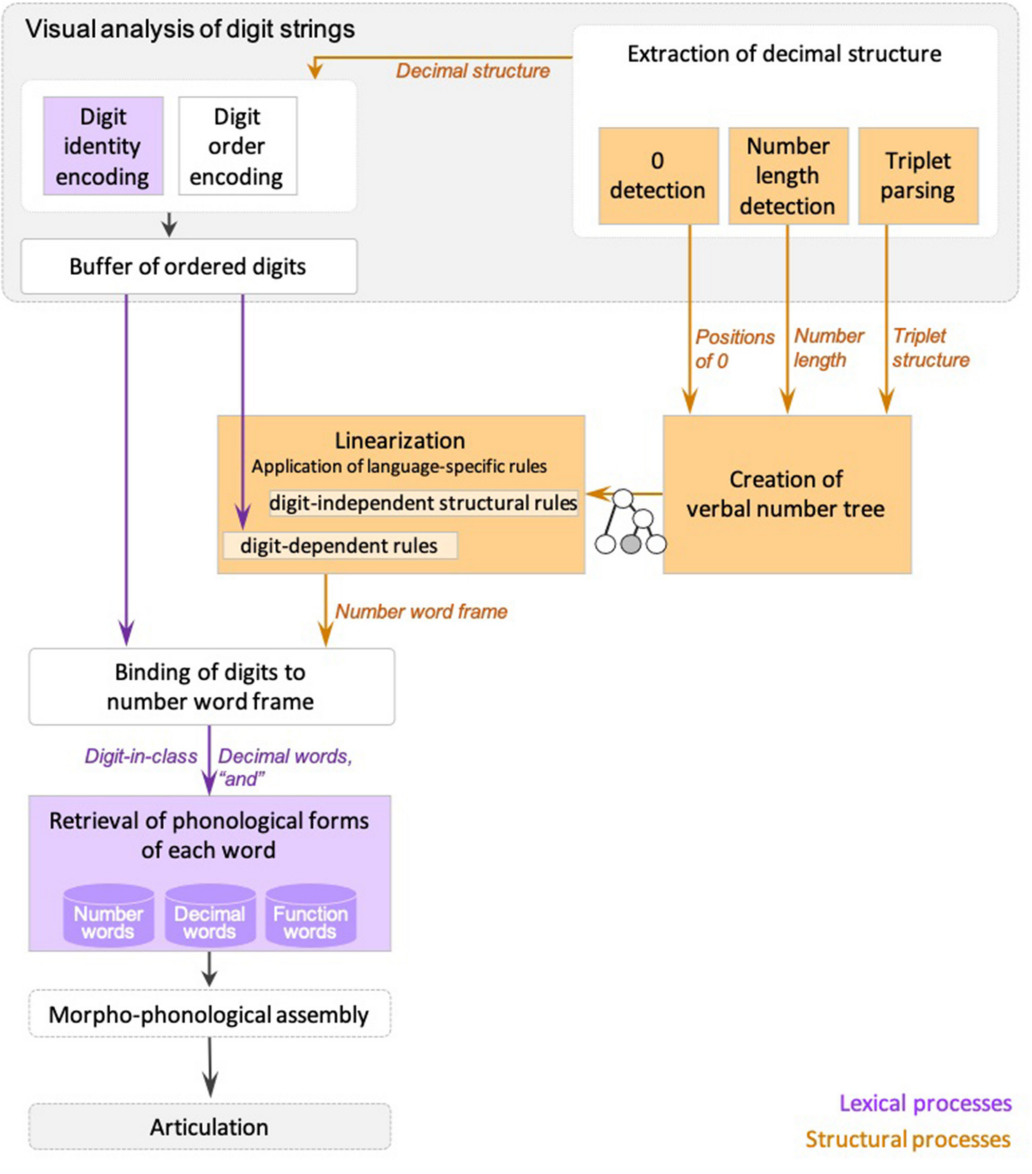
The number reading model from Dotan and Friedmann (2018).

Dotan and Friedmann (2018) proposed an intergrated model for reading aloud of numbers (depicted in Figure 1), which combines elements from the triple-code model (Dehaene, 1992; Dehaene and Cohen, 1995; Dehaene et al., 2003), and the number-reading models by McCloskey and colleagues (McCloskey et al., 1986; McCloskey, 1992) and Cohen and Dehaene (1991), and refines them based on findings from neuropsychological case studies of specific number impairments.

According to the model (Figure 1), multidigit number reading begins with numeric-visual analysis of the written number, which includes separate mechanisms for encoding of digit-identity, digit-order, and the extraction of decimal structure. The process of decimal structure extraction includes separate mechanisms for encoding of the number-length, detection of zeros and their positions within the number, and parsing of triplets.

The information about digit-identity and digit-order is then held in a dedicated numeric buffer as ordered digits, and the information on the decimal structure is sent to components responsible for creation of the number-word frame—the verbal form of the number. First, a syntactic tree is built, which is a hierarchical representation of the number (for example, 3-digit numbers are represented by a node for tens, a node for ones, and a higher node that merges the two, and by a hundreds node which merges with this smaller tree by an even higher node). The structure of the syntactic tree is determined by the way a language organizes verbal numbers into groups—e.g., in English and Hebrew numbers are organized into triplets. The tree is not influenced by language-specific order of number-words or irregularities unique to the specific language, which are taken into account at later verbal stages. Importantly, in Dotan and Friedmann's (2018) view, the syntactic tree is a part of the *verbal* representation of the number, rather than a general, abstract representation.

For reading aloud of the number, the constructed hierarchical tree is then *linearized* to the number-word frame by a set of language-specific conversion rules. In this stage, the properties of the specific language and the verbal form of multidigit numbers in it are taken into account—such as the order of number-words and the special rules concerning structure-modifying digits (e.g., 1 in the tens' position), which have an effect on the word frame.

Up to this stage—the number-word frame specifies slots for decimal words (such as the word “thousand” in 42,037) and function words (such as “and”), but still does not specify the number-words themselves (such as “two,” “seven”), only their lexical decimal classes. The abstract identity of the number-words is bound into the number-word frame in the next stage, which merges the ordered digits from the numeric buffer into the number-word frame produced in the linearization process. The result of this binding stage is a fully specified, yet abstract (not yet phonologically specified) sequence of words.

In the following stage, the phonological forms of the number-words, the decimal words, and the function words are retrieved from the dedicated phonological storage of number-words (in the phonological output buffer, Dotan and Friedmann, 2015. This buffer is a short-term component that holds ministores of phonemes, morphological affixes, and number-words). This sequence of words undergoes morpho-phonological assembly (the buffer is also responsible for assembling these units), which is then sent to articulation.

Deficits in each of the components of this multi-staged process, or in the connections between them, lead to various types of dysnumeria (McCloskey et al., 1985, 1986, 1990; Temple, 1989; Noel and Seron, 1993; Cipolotti and Butterworth, 1995; Cipolotti et al., 1995; Cohen et al., 1997; Basso and Beschin, 2000; Deloche and Willmes, 2000; Delazer and Bartha, 2001; Dehaene et al., 2003; Cappelletti et al., 2005; Friedmann et al., 2010; Starrfelt et al., 2010; Starrfelt and Behrmann, 2011; Moura et al., 2013; Dotan and Friedmann, 2015). Next, we will describe various types of dysnumeria that result from impairments to different components and their properties.

### 1.2. The Dysnumerias: Selective Deficits in Reading Numbers

Dysnumerias in the early stage of numeric-visual analysis—in the extraction of digit-identity, digit-order, or in the extraction of the written number's decimal structure were reported for several individuals, with developmental or acquired impairments.

***Digit-order dysnumeria*** is a deficit in the numeric-visual analysis component that encodes the relative order of digits in a multidigit number. People with this dysnumeria make digit-order errors in reading multidigit numbers, both in tasks that require verbal output and in tasks involving only silent reading, but not in phonological output tasks that do not involve written numbers (e.g., repetition of numbers). Dotan and Friedmann (2018; see also Friedmann et al., 2010) reported on two such women, EY and HZ, who made many digit-order errors in reading aloud and in silent reading but not in tasks that involved number production without numeric-visual input. YS, reported in Friedmann et al. (2010), may also have had this kind of dysnumeria.

***Numeric input buffer dysnumeria***affects the numeric input buffer without affecting the earlier stages that encode the digits and their order. Such deficit causes digit errors (substitutions, omissions), as well as digit-position errors, and may be susceptible to number-length effect. UN, reported in Dotan and Friedmann (2018) displayed such impairment (in addition to a deficit in number-word frame generation).

A specific deficit may also exist in the ***connection between the numeric input buffer and*** ***the later binding stage***, which may cause digit-identity errors in reading Arabic numbers aloud. This may be the deficit of YM, reported by Cohen and Dehaene (1991), and of BAL, reported by Cipolotti et al. (1995). BAL made “lexical” (digit-identity) errors in tasks that required reading Arabic numbers and producing them as spoken number-words. In contrast, his comprehension of Arabic numbers was intact, and he made no errors in the production of numbers written as number-words—suggesting that his deficit was neither in numeric-visual stages nor in phonological output stages. It seems, therefore, that BAL's deficit was in the connection of the written Arabic number to the number-word.

Going back to the early numeric-visual analysis stage, now to deficits in the decimal structure extraction components, ***Number-length dysnumeria***is a deficit in numeric-visual analysis, selectively affecting the function of encoding the length of the number. Individuals with this impairment make first-digit decimal shifts in multidigit numbers (e.g., reading the number 4320 as 43200). The deficit affects reading aloud as well as silent reading tasks that require processing of number-length (e.g., same-different decision task with numbers differing in length), but does not affect tasks that involve number output with no written input (such as multidigit number repetition). Such patients were reported by Dotan and Friedmann (2018): MA, who had a selective deficit in number length and did not make any other numeric-visual analyzer errors, and HZ (who had zero detection dysnumeria, in addition to her number length dysnumeria). An interesting manipulation used by Dotan and Friedmann, which we used in the current study as well, was the introduction of comma separators to multidigit numbers: when individuals with number-length dysnumeria read numbers with a comma separator, their decimal error rate decreases.

***Zero-detection dysnumeria***is a deficit in the zero-detector in the numeric-visual analysis stage of exctrating the decimal structure. This dysnumeria causes errors of zero omissions and additions (manifesting as decimal shifts) as well as transpositions of zero with meaningful digits. If this dysnumeria is selective, no decimal shifts are expected in numbers without zero. A patient who has this dynumeria is HZ (Dotan and Friedmann, 2018), who made digit-order errors in numbers including zero (and also, to a lesser degree, in numbers without zero, as she also had a deficit in digit-order).

Decimal shifts may also stem from a deficit in a stage that follows number-length encoding, which uses the number-length information for the creation of the decimal structure of the number. Individuals with ***a deficit in the generation of the decimal structure of*** ***written multidigit numbers***may perform well on tasks that require number-length encoding but still have a selective deficit in parsing the correctly-perceived number-length into triplets, for example, and may fail in the construction of the correct decimal structure of the number. This dysnumeria affects tasks of reading aloud but does not affect tasks that involve phonological output without written-number input such as number repetition. A participant who showed this dysnumeria was ED (Dotan and Friedmann, 2018, 2019). She made many first-digit decimal shifts in number-reading, performed well on visual-analyzer tasks that require processing of length, and benefited from reading the numbers with a comma separator, which did the job of parsing into triplets for her. When she was requested to look at a number without a comma and read it triplet-by-triplet (reading 654321 as “654 and 321”), she still made a similar rate of errors as when she read the number as a whole number, suggesting that she also had a difficulty in parsing into triplets, which might be a part of the decimal structure building. Dotan and Friedmann (2018) ascribed her deficit to a triplet-parsing component in the visual-analysis stage. In the revised model we suggest below, it can be described as a deficit in the conversion of the decimal information from the numeric-visual analyzer to the stage of decimal number frame generation, which may also include information about the division of the number frame into triplets. ED's sister, NL (also reported in Dotan and Friedmann, 2018, 2019), also showed a deficit in this stage (as well as in a later verbal production stage).

Stages of creating the number-word frame may also be susceptible to specific kinds of dysnumeria. The stages that follow the decimal structure extraction in the numeric-visual analysis are the generation of a number frame and the linearization of this number frame onto a verbal sequence. Due to this architecture, people with deficits in these stages show difficulties in reading aloud written numbers, but do not show difficulties in tasks involving the numeric-visual analyzer alone. Dotan and Friedmann (2018) report on several patients who were impaired in these stages: OZ and UN made decimal shifts in tasks involving number production, but not in tasks involving the numeric-visual analyzer alone. They made decimal-shifts both in reading aloud of multidigit numbers, and in tasks requiring production without visual-analysis (e.g., verbal responses to multipliciation and division problems, and UN also made such errors in repetition of multidigit numbers). Such deficit could have resulted from a deficit in the stage of number frame generation and linearization, or in later phonological stages. These shifts occurred mostly in the first digit, rather than in all positions, which excludes the possibility that they resulted from a deficit in phonological retrieval. Dotan and Friedmann (2018) concluded that OZ and UN's deficit is in the ***generation of the verbal syntactic tree or in the stage of linearization into a verbal*** ***sequence***.

A somewhat similar type of deficit was the one of ZN, an aphasic patient reported in (Dotan et al., 2014). ZN's deficit manifested itself in reading aloud of two-digit numbers. Dotan and Friedmann (2018) ascribed his deficit, like the deficit of UN and OZ, to either tree generation or linearization.

For our discussion below it is important to remark that Dotan and Friedmann's (2018) model takes both these stages—tree generation and linearization—to be verbal. Because ZN's comprehension of written two-digit numbers was spared—Dotan et al. (2014) and Dotan and Friedmann (2018) concluded that the comprehension of numbers does not require conversion of the multidigit number to its verbal representation, and that comprehension relies on a separate route of building the decimal representation.

However, there might be another approach to these results (suggested by the performance of the case study we report below). It might be that the generation of the number tree (or at least of the decimal number frame) is non-verbal, creating an abstract representation of the decimal structure of the number. Such abstract representation may be part not only of reading aloud but also of number comprehension. In this case, ZN's spared comprehension could be explained by placing his deficit in the verbal linearization stage, rather than in the abstract number frame generation stage.

A different locus, then, can be ascribed to the deficit of NR, reported by Noel and Seron (1993). NR had decimal shifts, most prominently in reading 3-digit numbers, with much fewer errors when only numeric-visual analysis was required (same-different length judgement, and the detection of the number of digits in a number, which can be performed in the visual stage, without comprehension). However, in contrast to ZN, NR's deficit also affected her comprehension of written numbers: she read and understood 3-digit numbers as if they were 4-digit numbers (e.g., 458->4508). NR's deficit can be attributed to the abstract number frame generation stage we have just proposed, which is shared by reading and comprehension, or to the connection between the numeric-visual analysis and the number frame generation.

Patient AT, reported by Blanken et al. (1997), made digit-order errors between the tens and the units digits in reading numbers aloud, whenever the German inversion rule (in which units are pronounced before tens) had to apply, but performed flawlessly in comprehending these numbers. It seems, therefore, that he had ***digit-binding dysnumeria***—a deficit at the stage of binding the digits with the decimal frame.

***Impairments in***later stages of ***the phonological production of number-words***cause number identity errors and decimal errors (substitution of numbers of different decimal classes) whenever verbal production of numbers is involved, but not in tasks of silent reading that only involve the numeric-visual analysis and possibly also number comprehension. Such cases were reported in Cohen et al. (1997), and for HY and JG in McCloskey et al. (1986). Dotan and Friedmann (2015) also reported on patients who had deficits in number-word production that resulted from a deficit in phonological output stages—a selective deficit in the phonological output buffer alone (SZ, GE) or also in the phonological output lexicon (YL, ZH, RB, and ZC). Impaired production of multidigit numbers due to phonological output deficits were also reported for GS (Girelli and Delazer, 1999; Delazer and Bartha, 2001) and for FA and RA (Marangolo et al., 2004, 2005).

Beyond the phonological output of numbers, some studies also reported selective impairments in writing of number-words. For example, patient BO (Deloche and Willmes, 2000) showed a selective ***impairment in number-word writing***. When presented with written multidigit Arabic numbers, she was able to read them aloud but failed to write them as number-words.

### 1.3. Why Test Dysnumeria in Sign Language?

These reported cases of specific dysnumerias show that specific stages of the number-reading process may be selectively impaired and cause different patterns of errors. In the current study we examine how a selective dysnumeria manifests itself in a different modality: in sign language. In addition to revealing how different types of dysnumeria may manifest themselves in a different modality, testing number-reading in sign languages has some unique advantages. First, it allows for a minimal comparison between reading of the same multidigit numbers with and without decimal structure: In ISL, multidigit numbers denoting quantity, such as number of objects, are signed with a decimal structure. However, a parallel system of non-decimal numbers exists which is used for signing numbers such as height, age, or bus numbers, and these numbers are signed as a sequence of digits, with no decimal structure (similar to the digital strategy used in some rural sign languages, as reported in Zeshan et al., 2013, and in Lingua dei Segni Italiana [LIS, the Italian sign language] for numbers 21–99, as reported in Mantovan et al., 2019, and as mentioned in Semushina and Mayberry, 2019, for ASL). Such non-decimal numbers (as do decimal numbers) are often signed in ISL in such a way that the digits are signed slightly moving from left to right locations (in right-handed signers), just like the direction in reading and writing written numbers.

Given a deficit in the creation of the decimal structure of multidigit numbers, as we claim our participant has, the existence of the non-decimal system allows for a direct comparison of the participant's signing of the same written multidigit number, once with a decimal structure and once without. Such comparison can provide an important view on the process of multidigit number reading.

Moreover, since sign languages do not have an orthographic system (Mayer, 2017), signers usually learn to read the orthography of the surrounding spoken language. Numbers written as number-words in the surrounding language provide the signer with a number frame, but in a different language and, in fact, a different modality –without providing clues about the verbal number frame in the sign language. This enables testing whether providing a number frame without clues about the required sign-phonology helps in reading numbers aloud. These two unique properties of sign languages can help to disentangle impairments specific to the construction of the number frame from impairments in other stages of the number-reading process.

As we will show below, the case of dysnumeria in sign language on which we report in this study will help in further developing the model suggested by Dotan and Friedmann (2018)—and in expanding it to also include stages required for the comprehension of multidigit numbers and to digit-by-digit reading.

### 1.4. Signing Numbers in ISL

**Figure 2:**
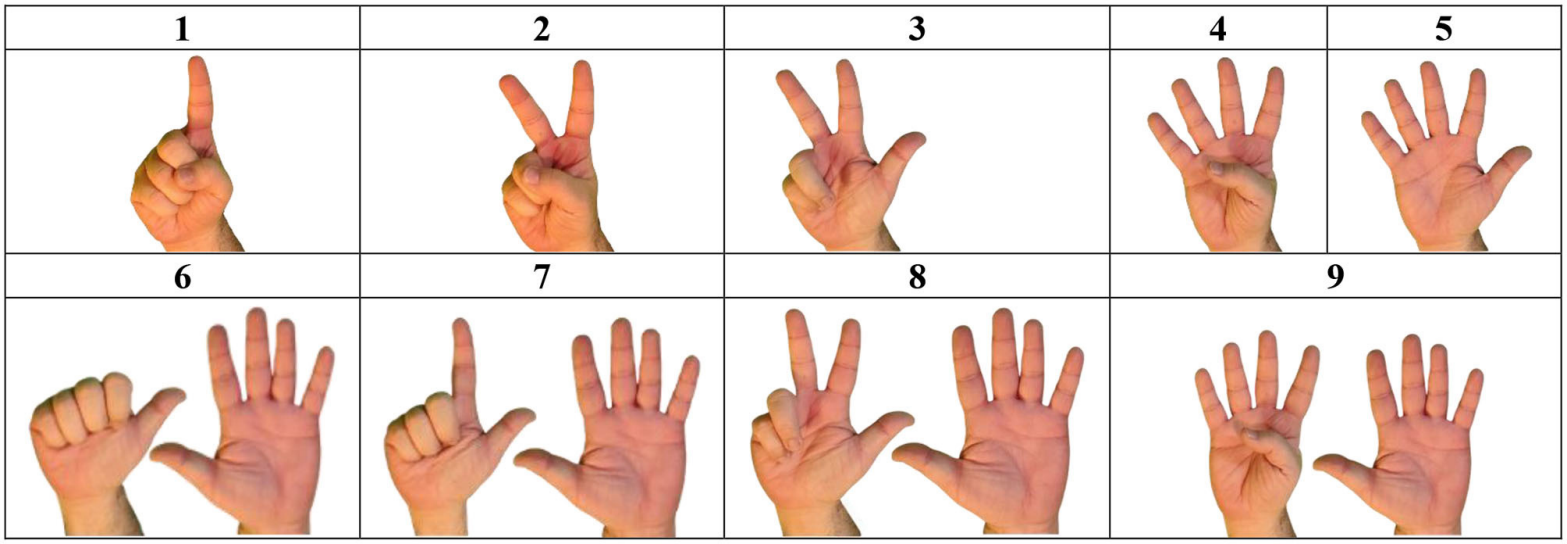
Numbers 1–9 in ISL illustrating phonological similarities between number-signs (e.g., the similarity between 3-8 and 4-9. See **Appendix** for variants of some of the number-signs).

**Figure 3:**
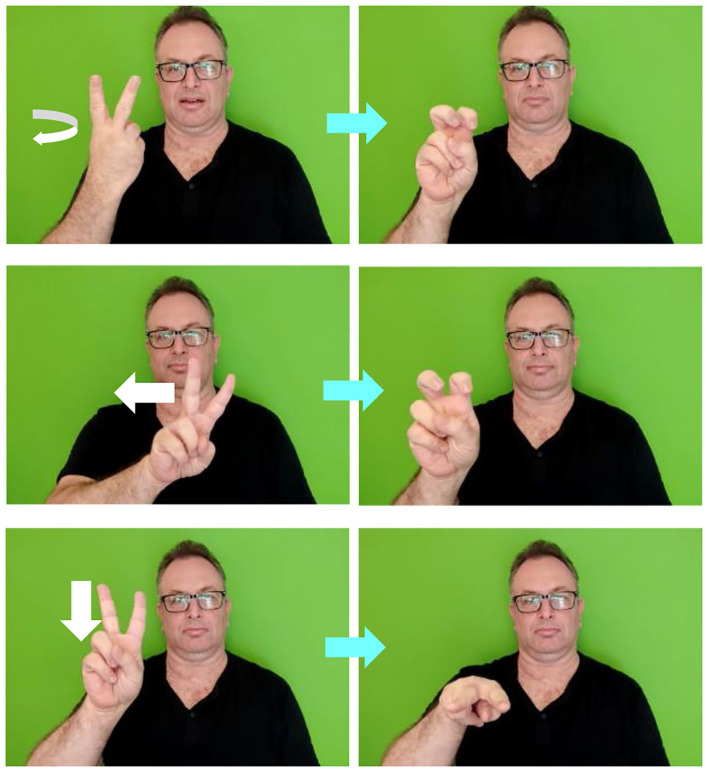
Decimal number-signs in ISL. Top: TWENTY, middle: TWO-HUNDRED, bottom: TWO-THOUSAND. (To see the videos, click the arrows).

ISL has a set of handshapes representing digits, as shown on Figure 2. In multidigit numbers in ISL, the decimal class of all but the unit digits is morphologically marked: the digit handshape is incorporated with the movement representing the decimal class, which together create the number-sign for this class. For example, as presented in Figure 3—in the ISL sign TWENTY, the handshape representing the digit 2 is incorporated with the movement representing tens, and in the sign TWO-HUNDRED, the handshape representing the digit 2 is incorporated with the movement representing hundreds.

**Figure 4:**
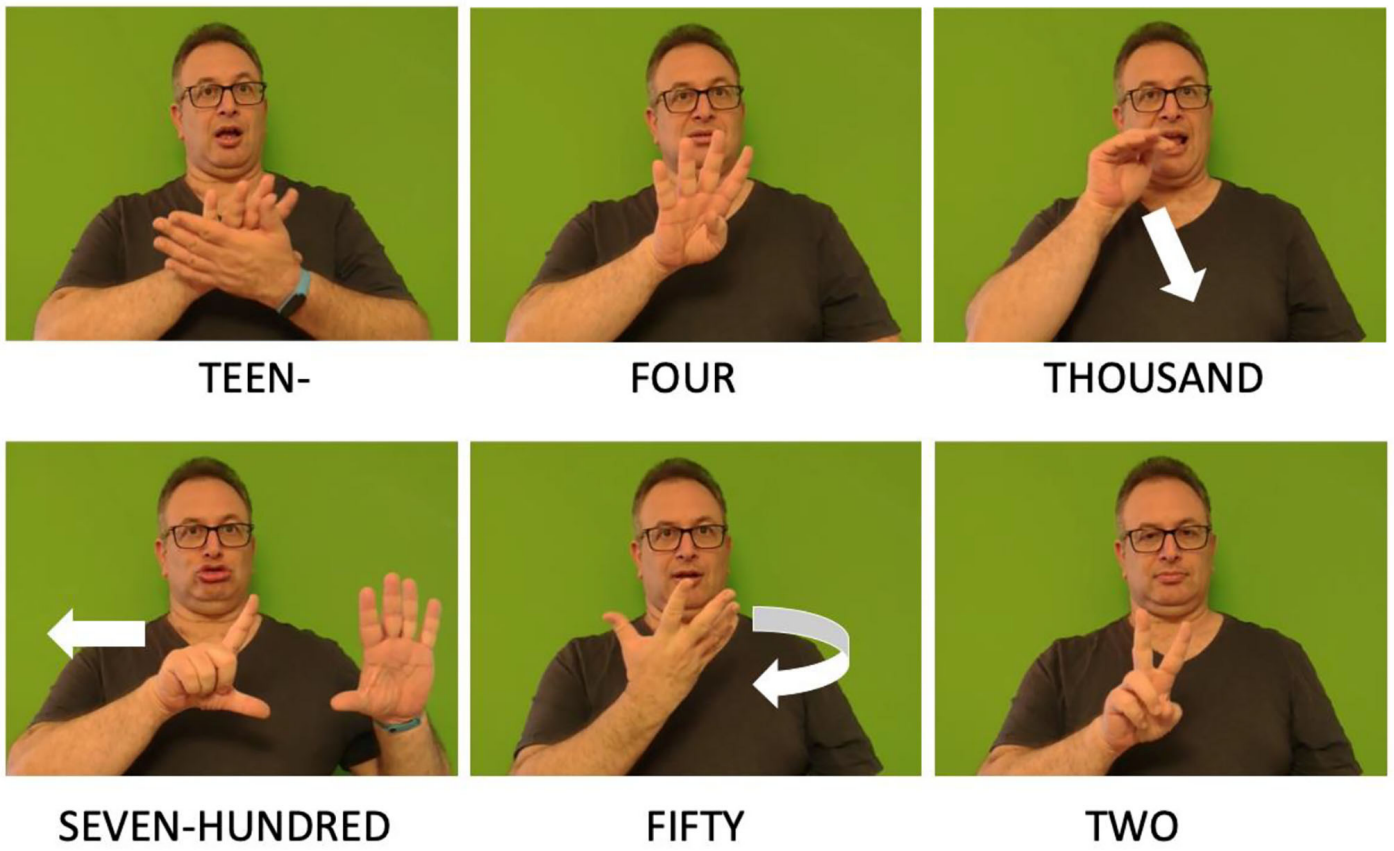
The multidigit number 14752 in ISL (To see the videos, click the pictures).

As in many other languages, such as English and Hebrew, multidigit numbers in ISL are organized into triplets—for example, the number 985672 is signed as NINE-HUNDRED EIGHTY FIVE THOUSAND, SIX-HUNDRED SEVENTY TWO. An example of a multidigit number in ISL can be seen in Figure 4.

ISL also has a unique structure for teens, but unlike many other languages (e.g., English, Hebrew), the part denoting teens is signed prior to the part denoting the specific units digit (i.e., TEEN-FOUR), so the tens and the units are still signed in the order in which they are written in the Arabic number (see Figure 4).

As can be seen on Figure 2, some pairs of digit signs in ISL (i.e., 8–3, 9–4) differ only in the use of the non-dominant hand. ISL users sometimes omit the non-dominant hand during signing, and use mouthing (moving the lips according to the parallel Hebrew word) to disambiguate the sign (e.g., 8 can either be signed with two hands- the dominant hand signing THREE, the non-dominant hand held with fingers spread, palm to the interlocutor, or it can be signed only with the dominant hand signing THREE, and the lips articulating “shmone,” the Hebrew word for eight). This phenomenon of non-dominant hand omission was probably boosted by the use of cellphones which are held in one hand, leaving only one hand for signing (during video calls, but also simply when the hand is occupied with holding the phone). There are also cases of hand addition, where 3 is signed like 8, which mainly occurs in the context of a neighboring two-handed number sign in which the non-dominant hand is kept in the “5” shape.

## 2. The Participant

Nomi is a 45-year-old woman, who is congenitally deaf and uses sign language as her main means of communication. As a daughter of hearing parents, she did not use sign language from birth, but was occasionally exposed to ISL from her deaf signing grandparents. She started to sign consistently only at a later age, and since she was 16, ISL is her main means of communication. Nomi had been using hearing devices since she was 1;6-year-old until the implementation of her cochlear implant around the age of 30 years, which she is using consistently when she communicates with hearing people since then.

Nomi told us that she always felt difficulty with numbers—and at school she was diagnosed as having a “learning disability,” with no further specification of her exact deficit.

We assessed Nomi's conceptual abilities using an odd-one-out task, in which she was presented with 30 sets of 4 pictures and was requested to select the one that is not related to the other pictures (e.g., three wild animals and a dog; three fruit and a vegetable, MILO test, Friedmann, 2017). Nomi performed perfectly (100% correct) on this task—indicating that her conceptual abilities are intact.

We also assessed her lexical knowledge in ISL, as well as her lexical retrieval components: the semantic lexicon, the phonological output lexicon, the phonological output buffer, and the connections between them, using a picture-naming task (SEMESH, Biran and Friedmann, 2004). In the picture naming task she was presented with 91 pictures and was requested to sign their names. Here again, Nomi performed flawlessly, with 100% correct responses– indicating intact lexical retrieval in ISL, including intact phonological output components, and rich lexical knowledge.

She also performed not differently from other ISL signers on a serial recall task of ISL digits (adapted from the FriGvi test battery, Friedmann and Gvion, 2002; Gvion and Friedmann, 2008), and even in the higher range of the control performance in serial recall of ISL signs (long sign span test from the SIMBA test battery, Haluts and Friedmann, 2019). Her digit span was 4 (performance of controls—signers without WM deficit: *n* = 10, mean = 5.15, SD=0.85) and her long sign span was 4.5 (controls: *n* = 17, mean = 4.4, SD = 0.6).

Her performance in the serial recall task indicated that her phonological working memory abilities, including the phonological input and output buffers, are intact, and the long-sign span also confirmed that her lexical knowledge of ISL is comparable to other ISL signers, as also indicated by her performance in the naming task and her word-reading-then signing task reported below.

## 3. General Method

We administered the tests detailed below to Nomi in six video (Zoom) sessions, each session was 1–2 h long. The sessions were video-recorded and analyzed and scored by all three authors separately. Nomi signed an informed consent prior to participation and was paid 50 ILS per hour. She was informed that she can stop her participation at any time and could take as many breaks as she wanted during the sessions. The research was approved by the Tel Aviv University Ethics Committee. All comparisons between Nomi's performance and the control groups were performed using Crawford and Howell (1998) *t*-test for the comparison of an individual with a control group, and the comparisons between Nomi's performance on the different tasks were done using McNemar exact or Chi-squared tests.

### 3.1. Control Group

Nomi's performance on the tests was compared to a control group of 10 deaf adults who use ISL as their main means of communication. They were tested in the same settings and procedure as Nomi did, in virtual Zoom sessions. Like Nomi, all of them have hearing parents and therefore did not acquire ISL from birth, but rather at a later age. They were 6 women and 4 men, aged 25–48 (mean = 32, SD = 6); nine of them were deaf from birth, and one gradually lost her hearing from birth until age 3. In the word-reading tests we also compared Nomi to control groups of typically-hearing native Hebrew speakers.

### 3.2. Error Analysis

Error analysis was conducted in the following way:

Substitutions of one digit with another (e.g., 589→579) were coded as *identity errors*; transpositions of digits that appeared in the number but were signed in an incorrect order (e.g., 3527 → 3257) and any production of a digit that appeared elsewhere in the number (985674 was signed as 96…) were coded as *order errors;*

Varieties that are used sometimes by ISL signers (and appeared also in our control group's signing), were not counted as errors. Therefore, additions and omissions of the non-dominant hand held in the “5” shape in signing a number were coded as acceptable. *Phonological errors* were other errors (not digit identity or number of hands errors) that involved the location, movement, or handshape of the digit (in effect, these errors were extremely rare, with a total of 3 for Nomi and for the control group together).

Errors in the decimal structure of the number (often regarded as “syntactic errors”) were coded as *decimal errors*. For example, had a participant started reading the number 45638 as 4563…, or made a decimal error resulting in an ungrammatical production (e.g., ‘forty fifty thousand…’, in which the decimal-class error was in the middle of the number), the error was coded as a decimal error. When the error resulted in shifting the decimal position of one or more digit, resulting in a decimal frame of an incorrect size, it was coded as a specific type of decimal error—*a decimal shift* (e.g., 80001→8001. Errors were coded as decimal shifts also when they were self-corrected at some point during the reading of the number 86952 → eight thousand, six hundred… ✔)^1^. We classified the decimal shifts according to the shift direction (whether it resulted in a smaller or a larger decimal class). Another type of error that was coded as decimal (and was much less frequent than shifts) was “thousand” omission—omitting the word/sign “thousand” between the triplets, which may suggest production of two separate triplets (with two smaller decimal frames) instead of the full number.

Finally, numbers for which the participant refused to sign and asked to move to another item were coded as “didn't sign” errors.

## 4. A Deficit in Multidigit Number Reading and Its Source

### 4.1. The Deficit: Reading Written Multidigit Numbers “Aloud”- Reading and Then Signing

#### Method

Nomi was presented with 60 multidigit numbers written one above the other (MAYIM battery, Dotan and Friedmann, 2014). Of these, 30 were shorter numbers (10 three-digit numbers and 20 four-digit numbers), and 30 were longer numbers (25 five-digit numbers and 5 six-digit numbers). The numbers of the various lengths were randomly ordered; 33 of the numbers did not include zero, and 27 included a single zero-digit. The task was similar to “reading aloud” task in spoken language: Nomi was asked to read each number and then sign it in ISL. We will henceforth relate to this test as “the baseline”.

#### Results

Nomi made errors on 18 of the numbers she read in this task (28%), and her performance was significantly lower than the controls' (mean correct = 91%, SD = 6%, Crawford and Howell's *t*(9) = 3.34 *p* = 0.004). Nomi's most pronounced error type was decimal errors, which occurred only in 5–6 digit numbers: she made 11 decimal errors (37% of the 5-and 6-digit numbers)—significantly more than the controls (mean = 6%, SD = 4%, Crawford and Howell's *t*(9) = 2.77, *p* = 0.01). For instance, when reading the number 89712 she signed “EIGHT-THOUSAND…NINE…NINE…NINE…,” and then corrected herself; When reading the number 985723 she signed “NINETY-EIGHT THOUSAND…, FIVE THOUSAND SEVEN HUNDRED TWENTY-EIGHT,” making a decimal as well as a digit-identity error. Most of Nomi's decimal errors were in the first, leftmost, digits, and the direction was always toward a smaller decimal position (89712 → ‘eight thousand…’ but not ‘eight-hundred ninety-seven thousand…'). She could not read correctly any of the 6-digit numbers, on which she either made a decimal error (on 3 of the 5 numbers in this length) or said that she cannot sign the number and asked to move on to the next one (2 numbers). She also made 3 non-decimal errors– 2 digit identity errors and 1 phonological error (which she immediately self-corrected). During the test Nomi reported that reading the long numbers is very difficult for her, and that she doesn't know how to sign them.

The next step was to try and find the origin of her deficit in reading multidigit numbers. Decimal errors in reading can arise from a deficit in the numeric-visual analysis of the written-number input, from a deficit in the conversion of written input- to the phonological (signed) output, or from a deficit in the phonological output processes.

### 4.2. Better Reading of the Same Multidigit Numbers as Non-decimal Numbers

To examine the source of Nomi's deficit in reading multidigit numbers, and specifically, to examine her numeric-visual analysis, we used a special property of ISL: some multidigit numbers that represent quantity (such as 61 seashells, or 123 new students) are signed as decimal numbers, with decimal morphology and structure, similar to numbers in spoken languages (e.g., 123 [students] is signed ONE-HUNDRED TWO-TENS THREE [the sign for 1 with hundred-morphology, the sign for 2 with tens-morphology, and the sign for 3]). However, a parallel system of multidigit numbers exists, which is used mainly for numbers that do not symbolize quantity (e.g., social-security numbers, bus numbers) and for certain measurement units (e.g., height, weight, age), in which the numbers are signed as a sequence of digits without a decimal structure, and without decimal morphology. For instance, when signing the (old) age of 123, the number will be signed “ONE TWO THREE,” with no decimal structure.

This allowed us to isolate the conversion to a decimal structure from the other components of multidigit number reading: visual-analysis of the sequence of digits and phonological production of the sequence of number-signs, and compare Nomi's signing of the exact same multidigit numbers with and without the conversion to a decimal template.

#### Method

Nomi was presented with 60 numbers, same numbers as in the baseline task (section 4.1), presented in the same way. The only difference was the instruction: now she was told it is a list of passwords, and she was again asked to read and sign.

#### Results

Once she was requested to sign the numbers without their decimal structure, Nomi was far more accurate. She commented that signing numbers this way was much easier for her than signing them as quantity numbers. She signed with confidence, and, like the control group- from left to right. She made only 2 digit-order errors (transpositions of adjacent digits), so her performance was significantly better than her reading of the exact same numbers with a decimal structure (97% correct), McNemar test *p* = 0.0001 (one-tailed).

Nomi's performance in this test was comparable to that of the controls' (mean = 98%, SD = 2%, Crawford and Howell's *t*(9) = 0.80, *p* = 0.22).

Her far-better performance on reading the same numbers when she did not need to convert them to decimal structures indicates that her visual-input processes themselves are intact and cannot be the source of her deficit. Had she had a deficit in the perception of number-length or in the position of zeros, we would have expected similar errors in reading digit-by-digit—errors of omission or doubling of digits, or errors in the position of zero, which she did not show. This finding, suggesting preserved visual analysis of number-length, together with the decimal errors she made in reading the same numbers decimally, hint at a deficit related to the decimal structure, in a stage later than the numeric-visual stage.

### 4.3. Presentation of 6-Digit Numbers With an Instruction to Read Each Triplet Separately

If Nomi's impairment is indeed in converting number-length of long numbers into number frames, another manipulation expected to help her in reading long numbers is reading the number triplet-by-triplet, breaking the long number into two shorter 3-digit decimal numbers (see Dotan and Friedmann, 2019, for this manipulation in dysnumeria).

#### Method

Nomi was presented with 20 6-digit numbers (all with 6 unique digits) and was requested to split the numbers in two triplets such that she read the first 3 digits as one decimal triplet, and the last 3 digits as another decimal triplet (e.g., when presented with 123456, she was expected to sign ONE-HUNDRED TWENTY-THREE; FOUR-HUNDRED FIFTY-SIX).). Here we provided her both with the length of the number (as there were 6 digits in all numbers presented in this task), and exempted her from the need to sign numbers >999, namely, she did not need to construct a tree higher than hundreds and did not need to produce thousand, 10-thousand, or 100-thousand number-signs.

#### Results

Nomi did not make any errors in this test and signed correctly all of the triplets (100%). She also used mouthing of the Hebrew decimal number correctly in all of her productions. This suggests, again, that when she does not need to create a number frame higher than 3-digits, Nomi shows no difficulties. Her performance in this task thus further supports the idea that her deficit arises when she needs to create a decimal number frame for numbers longer than 3-digits.

### 4.4. Additional Evidence for Intact Numeric-Visual Input Processes: Three Same-Different Tasks and a Sequence Decision Task

Nomi's decimal errors in reading multidigit numbers could have arisen from a deficit in the input stage of numeric-visual analysis, in processing number-length or digit position; alternatively, it could stem from a deficit in creating a number frame that would match the written number. Her good performance in the digit-by-digit and the triplet-by-triplet reading of multidigit numbers, described above in sections 4.2 and 4.3, indicates that she did not have a digit-order deficit in the numeric-visual input stage (nor did she have a deficit in digit-identity, but this component anyway is not a candidate for decimal errors). It is a bit trickier to guess how a number-length deficit would manifest itself in digit-by-digit reading: it could cause digit-omission, zero insertion or omission, and perhaps digit-duplication. Nomi did not make such errors in the digit-by-digit task and the triplet-by-triplet task. To further examine Nomi's visual input processes of number-reading, and to examine directly her numeric-visual encoding of number length and order, we tested her in four tasks that involve the numeric-visual analysis of written numbers, without requiring production.

#### 4.4.1. Same-Different Tasks

Since same-different decision tasks of written Arabic numbers involve only numeric-visual input, and do not require any verbal output, nor do they require the creation of a number frame, they allow for a direct and specific examination of the numeric-visual analyzer. In the same-different tasks we examined the encoding of number-length, digit-identity, and digit-order, by way of manipulating the differences between the two numbers—they could either differ in length, identity, or order.

To manipulate number-length without the concomitant manipulation of identity and order, we used the duplication of digits (e.g., deciding whether 9939 and 99399 are the same). This allowed for the specific and direct examination of length extraction in the numeric-visual analyzer, since the numbers differing in length do not also involve a change in digit-identity (they contain the same unique digits), neither do they involve a change in relative digit-order (the digits are written in the same order, with an additional duplicate in one of the number's ends). This allowed us to examine whether Nomi's decimal errors result from a deficit in length extraction in the numeric-visual analyzer.

##### 4.4.1.1. Same-Different Decision: Pairs of Numbers Presented Together

Nomi's ability to detect differences between written numbers was first tested in a same-different task in which the pairs of numbers were presented on a screen together, one above the other, without time limitation. Nomi was requested to judge, for each pair, whether the numbers were identical or different. The task included a total of 118 number-pairs, all including a repeating digit and another digit, presented in two sessions: 27 pairs of 3–6-digit numbers that differed in length (created by the addition of another instance of the repeating digit, e.g., 99399 and 9939), 20 pairs that differed in order (e.g., 99899 and 98999), 16 pairs that differed in number-identity (e.g., 97999 and 95999), and 55 identical pairs.

##### 4.4.1.2. Same-Different Decision: Numbers Presented Sequentially

To rule out the possibility that Nomi succeeded in the first same-different task because the comparison of simultaneously presented pairs was too easy, we also administered a similar task with delayed comparison, where the numbers of each pair were presented on a computer screen one after the other (using Testable). The first number of the pair was presented for 1200 ms, followed by a 500 ms masking period, and then the second number appeared for 1200 ms. Then a question mark appeared on the screen and the participant decided whether the numbers in the pair were the same (by pressing the “l” key) or different (by pressing “a”). This task included a total of 40 pairs: 10 pairs of 3–6 digits numbers that differed in length (7 of which were pairs of 4–5- and 5–6-digit numbers), 8 pairs that differed in number identity, and 22 identical pairs.

##### 4.4.1.3. Number Matching (99499)

Another way in which we examined Nomi's number-reading when no verbal production is required was a matching task. Nomi was presented with a page on the screen, on top of this page appeared a reference target multidigit number and 36 numbers printed underneath. She was requested to circle all numbers that were identical with the reference number, as accurately and quickly as possible.

The reference number was 99499, and the 36 numbers beneath it included 5 items that differed from the reference in digit-order (by transposing 9 and 4), 25 that differed from it in length (by adding or subtracting instances of the digit 9), and 6 items that were identical to it.

##### 4.4.1.4. Same-Different Tasks Results

Nomi showed very high performance on all three same-different tasks, as summarized in Table 1. When presented with the pairs written one above the other (4.4.1.1), she made no number-length, digit-order, or digit-identity errors at all, and had only 2 misses of identical pairs that she did not mark (4% of the identical pairs).

**Table 1:** The number tests and Nomi's performance in them.

**Test (Section)**	**Description**	**# items**	**% correct**	**Decimal errors**	**Other errors**
Reading and signing (RS) multidigits-the baseline task (4.1)	Read-then-sign multidigit numbers written as Arabic numerals	60	70%	11 decimal errors in 5–6 digit numbers, 2 “don't know” errors in 6-digits.	2 identity errors and 1 phonological error (which was immediately self-corrected)
RS multidigits as non-decimal numbers (4.2)	Read-then-sign same numbers as in the baseline task, but this time digit-by-digit	59	90%	–	2 serial order errors with immediate corrections
RS Multidigits—Triplet-By-Triplet (4.3)	6-digit numbers written as Arabic numerals triplet-by-triplet	19	100%	No decimal errors	No errors
Same-different multidigits: simultaneous (length, order, identity) (4.4.1.1)	Pairs of multidigit Arabic numerals presented simultaneously, asked to judge same/different	118 pairs	98%	–	2 misses of identical pairs
Same-different multidigits: sequential (length, identity) (4.4.1.2)	Pairs of multidigit numbers presented one after the other with masking between them, asked to judge same/different	40 pairs	98%	–	1 miss of an identical pair
Same-different multidigit matching (Length, order) (4.4.1.3)	Target multidigit number and 36 numbers below it, asked to mark numbers similar to target	36	94%	–	2 misses of matching numbers
Sequence decision in multidigits (4.4.2)	4-digit numbers written as Arabic numerals, mark the ones that contain a sequence of monotonic increasing consecutive digits	118	98%	–	2 order errors
RS multidigits written as number words (4.5.1)	Read-then-sign multidigit numbers written in Hebrew words	18	94%	No decimal errors	1 whole-unit error
Repetition of multidigits (4.5.2)	Repetition of multidigit numbers presented as ISL signs	45	42%	2 decimal errors	Many identity and order errors
Repetition of multidigits with fewer number-words (multiple zeroes) (4.5.3)	Same task as (4.5.2), with numbers requiring fewer number-signs	37	84%	No decimal errors	6 errors−4 identity errors, 1 order error, and 1 morphological error
RS multidigits with fewer significant digits (multiple zeroes) (4.5.4)	Read-then-sign same numbers as (4.5.3) written as Arabic numerals	40	60%	9 decimal errors (23%), and 6 “don't know” responses	1 digit identity error (immediate self-correction)
>5500—written input no comma (4.6.1)	Decide whether multidigit Arabic numerals are >5500	20	80%	4 errors: all 5-digit numbers in which the first digit <5	-
>5500—signed input (4.6.2)	Decide whether signed multidigit numbers are >5500	30	97%	1 error –first item	-
Multidigit number-comparison (4.6.3)	Decide which of a pair of multidigit numbers written as Arabic numerals is greater	68 pairs	97%	3 Errors in longer numbers. Much longer RTs than controls in same-length 6-digit pairs, and in different-length pairs with incompatible first digit.	-
RS multidigits with a comma separator (4.7.1)	Read-then-sign same numbers as in the baseline task (4.1), but with a comma separator	60	97%	2 decimal errors	–
>5500—written input with comma (4.7.2)	Same task as in written > 5500 (4.6.1), but with a comma separator	30	100%	–	No errors
Numeral incorporation (NI) (4.5.5)	Read-then-sign (translate to ISL) written Hebrew sentences containing NI phrases	42	100% on relevant structures		No errors in the NI structures

*Dark gray, significantly more decimal errors than the controls; Light gray, significantly more than the controls but much fewer than in the reading and signing baseline task*.

In the sequentially-presented numbers task (4.4.1.2), she made no number-length or digit-identity errors and had only one miss (pressing “different” for a same pair, 98% correct). This performance is not different from that of the control group (mean = 95%, SD = 9%, Crawford and Howell's *t*(9) = 0.33, *p* = 0.38).

In the number matching task (4.4.1.3), Nomi made no length or order errors– she never marked a number that was different from the reference number. She did miss 2 of the 6 identical numbers.

In all same-different tasks, thus, she never made any errors of mistakenly marking a different pair as similar, and importantly—she never mistook a pair of numbers *differing in length* as a similar pair.

In addition, Nomi explicitly reported that these same-different tasks were easy for her, she even used the sign for “fun.”

#### 4.4.2. Additional Evidence for Intact Numeric-Visual Input Processes: Sequence Decision

##### Method

Nomi was presented with 118 4-digit numbers (sequence decision task, MAYIM battery, Dotan and Friedmann, 2014), 60 of which were strictly monotonic increasing sequences of consecutive digits (e.g., 1234), 33 included a transposition between two adjacent digits, such that the serial sequence was violated (e.g., 1243), 25 included a substitution of one of the digits such that it did not create a monotonic increasing sequence (e.g., 1274). Nomi was asked to mark all the numbers in which the digits created a consecutive monotonic increasing sequence.

##### Results

In the sequence decision task, Nomi made only 2 errors (<2%)—both on the same sequence (5687) which she marked even though it was a transposed sequence. This indicated that her digit-order encoding is intact.

#### 4.4.3. Interim Summary: Assessment of Nomi's Numeric-Visual Analysis

Nomi's far better reading of multidigit numbers when she did not need to convert them onto decimal structures larger than a triplet, as well as her good performance on the same-different and sequence decision tasks all point together to the same conclusion: her numeric-visual input processes are intact and cannot be the source of her deficit. She did not have a deficit in number-length perception, nor in digit-order or in zero-position, which could be the basis for her decimal errors in reading-then-signing multidigit numbers. Her flawless performance in reading the same multidigit numbers digit-by digit indicates that her numeric-visual analysis of digit identity and order is intact. The locus of her deficit, then, has to be a later stage in the number processing model: either in the conversion of the written number into its decimal frame, in constructing the decimal structure of the number frame, or in later phonological output stages.

### 4.5. The Decimal Errors Do Not Stem From a Phonological Output Deficit

#### 4.5.1. Multidigit Numbers Written in Number Words

Had Nomi's deficit been in the number production stages in the phonological output buffer, responsible for retrieval and assembly of number-words, we would expect her to make errors not only when she reads multidigit numbers, but also when she produces them without reading. To test Nomi's production of multidigit numbers without reading of Arabic numerals, and in a way that provides her with the decimal structure of the numbers, we took advantage of her being Hebrew-ISL bilingual, and provided her with the decimal structure of the numbers in a separate system- a written spoken language, which does not provide her with the needed number-signs in ISL. We presented her with multidigit numbers written in Hebrew number-words (e.g., five hundred twenty-four; Word-To-Number test, MAYIM battery, Dotan and Friedmann, 2014). Notably, number signs do not show one-to-one correspondence to written number words (for example, “four hundred” is written as two Hebrew words but corresponds to a single number-sign). This allowed us to test Nomi's production of multidigit numbers once the decimal structure is provided, and thus tease apart the conversion of the written number to a decimal structure, and the production of the decimal structure of multidigit numbers.

##### Method

The test included 18 multidigit numbers written in Hebrew number-words. The target signed numbers were composed of 2, 3, 4, 5, and 6 number-signs (4, 5, 3, 5, and 1 items, respectively). Nomi was requested to read the Hebrew written words and, when she finished reading the whole number, sign the number in ISL.

##### Results

Nomi made no decimal errors at all in this test. She only made one whole-unit error (in response to the Hebrew number-words “three thousand and-nine teen” she signed “3900” (parallel to “three thousand and-nine hundreds”), and then corrected herself. This performance indicates that her deficit in the decimal structure of multidigit numbers does not stem from impaired retrieval of the correct number signs in the correct decimal morphology at the stage of the phonological output buffer, nor from a deficit in holding these signs and assembling them in this buffer, but rather from a component that converts the written Arabic numbers into the number frame.

#### 4.5.2. Repetition of Multidigit Signed Numbers

Another way to examine Nomi's production of multidigit numbers without involving written input of Arabic numerals was to ask her to repeat a list of numbers. These were the same numbers she had read on the baseline reading task (4.1), list on which she made many decimal errors.

##### Method

The task included 45 of the multidigit numbers from the baseline number reading task. The signed numbers included 3-, 4-, 5-, and 6-digits/ number signs (9, 12, 20, and 4 items per length, respectively). A native signer signed each multidigit number and Nomi was requested to repeat it immediately, as accurately as possible.

##### Results

It seems that the 4–6-digit numbers exceeded Nomi's signed digit span (which was 4, see Participant description) so she omitted and substituted digits in 75% of the 5–6 digit numbers she tried to repeat and in 50% of the 4-digit numbers, with a general percentage correct of 42%. However, crucially, her pattern of errors was completely different from the one she displayed in number-reading: she made only 2 decimal shifts in her repetition, both with correct Hebrew mouthing despite the manual error, significantly fewer decimal shifts than in reading the same numbers (McNemar test *p* = 0.02). She also had repetition-errors on shorter numbers than in reading: in 4- and even 3-digit numbers.

#### 4.5.3. Repetition of Signed Multidigit Numbers With Fewer Unique Digits and Fewer Number-Signs

##### Method

Nomi's high rate of non-decimal errors in repetition of multidigit numbers in the previous task might stem from exceeding her working memory capacity. We therefore tested her repetition of multidigit numbers in a task that was less taxing for her phonological working memory, which used multidigit numbers (3–6 digits) with fewer (3–4) significant digits and fewer number-signs (2–3). For example, 403000 is a 6-digit number, the same length as the longest numbers in the baseline list (4.1) section and Its Source, but it requires only 3 number-signs in production (four-hundred, three, thousand). This test included 7 three-digits numbers, 9 four-digit numbers, 18 five-digit numbers, and 6 six-digit numbers.

##### Results

In repetition of these numbers Nomi made no decimal errors at all. She did make 4 number-identity errors (e.g., substitutions of one number-sign with another), one order error (i.e., she repeated 736 as 763) and one error of number morphology (in repetition of the number 3021, she signed separate signs for “three” and “thousand” instead of the ISL single sign “three-thousand”). She reported at the end of the test that she was not focused, so this may be the cause of these errors. Importantly, here again the errors were not unique to longer numbers—half of them happened with 3–4-digit numbers, and, crucially, were not decimal errors.

#### 4.5.4. Reading and Signing Long Multidigit Numbers With Multiple Zeros

The last three experiments demonstrated that Nomi has no decimal structure problems in producing multidigit numbers once no written Arabic numerals are involved. In the next task we took an additional view as to the question of whether her decimal errors in number-reading (in the baseline task) stemmed from a phonological overload of number-signs. To examine this, we tested her reading of multidigit numbers of the same length as in the baseline task, but this time with fewer number-signs.

##### Method

We asked Nomi to read 40 multidigit numbers with fewer significant digits (3–4) and fewer number-words (2–3). The numbers included multiple zeroes instead, and were the same numbers used in the task reported in the previous section (4.5.3).

##### Results

Even though these numbers required far fewer number-words than did the numbers in the baseline list, Nomi still made many errors in this task (43%) and showed a similar error rate (χ^2^ =1.65, *p* = 0.20) to the one she showed in reading-then-signing numbers without multiple zeros (in the baseline task). This result indicates that her errors did not result from an overload of number-signs in the phonological output buffer, but rather have a different origin.

Her error pattern in this test was also similar to the one she showed in the baseline task: She made 9 decimal errors (23%), and refused to sign 6 numbers, remarking these were too long for her. Like in the baseline, the majority of her errors occurred in the longer numbers: she could not sign any of the 6-digit numbers (one decimal error and 5 refusals), and could sign only 56% of the 5-digit numbers (7 decimal errors and one refusal). She made only one decimal error in a 4-digit number, and no errors in the 3-digit numbers. Here again, as in the baseline task, the direction of decimal shifts was almost exclusively toward the smaller decimal position.

Nomi's performance in this test was significantly worse than her performance in the parallel repetition task (in the previous section), in which she was required to repeat exactly the same 40 numbers (McNemar test *p* = 0.01).

Just like when reading and signing the numbers without multiple zeros (in the baseline task), Nomi struggled in this test. She said that it was very difficult for her, and that she found the long numbers especially hard.

#### 4.5.5. Numeral Incorporation

**Figure 5:**
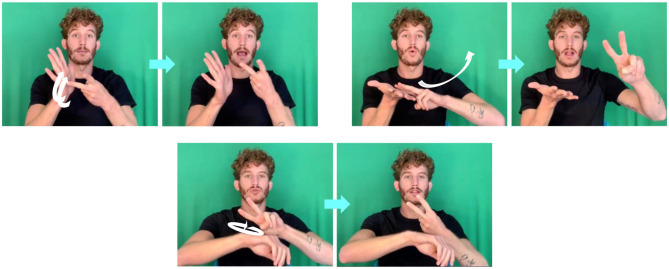
Numeral incorporation in ISL. Top: TWICE, middle: TWO-MONTHS, bottom: TWO-HOURS (To see the videos, click the arrow in each of the picture pairs).

Nomi's decimal errors were mostly decimal shifts, in which she produced an incorrect decimal class for the correct digit. In ISL, the decimal classes are marked morphologically on the number. Therefore, we wanted to rule out the possibility that the decimal errors originated in a deficit in morphological incorporation of numbers. ISL allows for a direct assessment of this question. As in other sign languages (Liddell and Johnson, 1989; Taub, 2001; Fuentes and Tolchinsky, 2004; Meir and Sandler, 2007; Fischer et al., 2011; Semushina and Mayberry, 2019), ISL uses morphologically complex structures in which a number-sign is incorporated into a base morpheme, usually denoting a time expression or a pronoun—to create a single morphologically-complex sign (e.g., EIGHT-YEARS is a single ISL sign made from the handshape of the number 8 and the movement and location of the sign YEARS, and THREE-OF-US is a sign made from the handshape of the number 3 and the movement and location of the sign US, see Figure 5 for some examples). If the decimal errors emerged from a morphological difficulty in numbers incorporated in morphologically-complex structures, these non-decimal morphological structures should be affected as well. Additionally, morphological incorporation takes place in the phonological output buffer (Haluts and Friedmann, 2020), so these constructions could also serve as another assessment of Nomi's phonological output buffer.

##### Method

To test Nomi's production of structures with numeral incorporation, we presented her with 42 written Hebrew sentences containing numbers−19 of which contained structures signed as numeral incorporation in ISL, and asked her to translate them into ISL.

##### Results

Nomi made no errors at all in the structures involving numeral incorporation. Her good performance on these structures, then, rules out a deficit in the complex morphology of numbers as the basis of her decimal errors, and points to a difficulty that is unique to multidigit numbers.

Additionally, Haluts and Friedmann (2020) showed that signers with impairments to the phonological output buffer make whole-unit errors in these morphologically-complex structures. The finding that Nomi performed well on these structures, as well as her consistent direction of errors - always toward a number which is smaller in one decimal-position, provides another support for the conclusion that her phonological output buffer is intact.

#### 4.5.6. Interim Summary: Assessment of Nomi's Phonological Output

The above tasks indicated that Nomi's decimal errors did not result from a deficit in phonological output processes of selecting the correct number signs including their correct decimal morphology, holding them, and assembling them into a whole multidigit number. She did not make any decimal errors in signing multidigit numbers written as Hebrew words, even though this task requires the same stages of phonological selection, holding, and assembly of complex number-signs. In repetition of multidigit numbers she made almost no decimal errors. When reading long multidigit numbers that require fewer number-signs in production (which reduces the load on the phonological output buffer) she still made the same rate of decimal errors. Finally, she showed normal production of numeral-incorporation structures, which shows that she can produce morphologically complex numerical structures. As we will show below, the addition of a comma-separator, a visual manipulation that does not affect phonological output, significantly reduced her decimal errors. Together with her intact numeric-visual analysis, these results point to a deficit in a decimal structure stage that follows visual input and precedes phonological output.

### 4.6. Same Impairment in the Comprehension of Written Multidigit Numbers

In the previous sections we have seen that Nomi makes decimal errors in reading and then signing multidigit numbers, and that this deficit cannot stem from her numeric-visual analysis or from the phonological output stages, which were intact. We suggested that the deficit is related to the creation of the decimal number frame for written multidigit numbers. The question now is whether the deficit only affects decimal structure required for phonological production, or whether the deficit also affects tasks that do not require phonological output, such as comprehension tasks.

#### 4.6.1. Impaired Comprehension of Written Numbers: > 5500

##### Method

To test Nomi's comprehension of multidigit numbers (in a test that does not involve production), we presented her with 20 multidigit numbers printed on one page, and asked her to mark all numbers that are greater than 5500. The reference number (5500) was given to her in signing. The multidigit numbers included numbers of different lengths (3–5 digits, 5 of them with 5 digits) without a comma separator, randomly scattered on the page. Importantly, the five 5-digit numbers, which, obviously, were all greater than 5500, included four numbers for which the first, leftmost digit was smaller than 5. If her deficit in decimal structure affects comprehension of written numbers in the same way it affects reading-then-signing, we would expect that she might apply to these numbers a 4-digit-number structure instead, and then may understand them as smaller than 5500.

##### Results

Of the five 5-digit numbers, Nomi marked only the one in which the first digit was >5 (93061), but missed all other four (80% errors), in which the first digit was smaller than 5 (e.g., 30901, 20302)—indicating that she did not comprehend the decimal structure of these longer numbers, and therefore estimated the size of the number only on the basis of the identity of the leftmost digit (e.g., when she read 30901, she may have created a 4-digit decimal frame for it, starting with three-thousand, and hence judged it as smaller than 5500).

Nomi had no errors with the 3- and 4-digit numbers. She made significantly more errors on this test than the controls (who made only 3% errors, SD = 7%, Crawford and Howell's *t*(9) = 2.40, *p* = 0.02).

#### 4.6.2. Intact Comprehension of Signed Numbers: >5500

We used the same task as in 4.6.1, but this time the numbers were signed to Nomi rather than presented in writing.

##### Method

The task included the same numbers as in the previous section (4.6.1), and additional 10 numbers of different lengths—a total of 30 numbers (13 of them were 5-digit numbers, and in eight of these the leftmost digit was smaller than 5). Nomi was presented with a signed multidigit number and was requested to decide, for each number, whether it was greater or smaller than 5,500.

##### Results

Nomi made only one error in this task, on the first number presented in the task (5601). She responded correctly to all other numbers, including for the eight 5-digit numbers in which the first digit was smaller than 5—the type of numbers that she missed in the written version of the test.

These two >5500 tasks together indicate that Nomi has a similar deficit in comprehension to the one she has in reading-then-signing when the numbers are written, but she does not have a deficit in understanding these numbers when they are signed rather than written to her. This suggests that it is not the comprehension processes themselves that are impaired but rather getting to these processes from written Arabic number input.

#### 4.6.3. Impaired Performance in a Number Comparison Task

We further assessed Nomi's comprehension of numbers using a number comparison task, in which she was requested to decide which of two multidigit numbers is greater.

##### Method

The task included 68 number pairs, in 40 of which the numbers differed in length, and in 28 the numbers were in the same length. Of the different-length pairs, 32 were such that the first digit of the longer number was *smaller* than the first digit of the shorter number (e.g., 6493 and 52879, henceforth: the “incompatible condition”). The incompatible condition included 16 pairs of a 4- and a 5-digit number, and 16 pairs of a 5- and a 6-digit number. Eight other pairs were of the ‘compatible’ condition, in which the first digit of the longer number was also *greater* than the first digit of the shorter number.

Of the same-length pairs, 20 (8 pairs of 4-digit numbers, 8 of 5-digit numbers, and 4 of 6-digit numbers) differed already in the first, leftmost, digit, and 8 (3 pairs of 4-digit numbers, 3 of 5-digit numbers, and 2 of 6-digit numbers) differed only starting the second or third digit.

The numbers in each pair were presented to Nomi written one next to the other with 5 spaces between them. She was requested to decide, for each pair, whether the right or the left number was greater by pressing either the right or the left arrow on the keyboard. The pair remained in front of her until she pressed a key, without time limit. The test began with four practice items.

##### Results

We did not press Nomi for response times in this task, and her RTs were generally longer than those of the control participants, though not significantly (Nomi: M = 3003 ms, SD = 1,490; control M = 2188 ms, SD = 703; RT for error responses and outliers of >3SD from the participant's mean and RTs for errors were excluded). This probably resulted in her not making many errors (she made 3 errors, all of them in the longer numbers: one in a pair of 5- and 6-digit numbers of the “incompatible” condition, and two in pairs of 6-digit numbers with a different first-digit. The number of errors she made was significantly larger than that of the controls (M = 1.30, SD = 0.82, Crawford and Howell's *t*(9) = 1.98*, p* = 0.04), but still relatively low.

**Figure 6:**
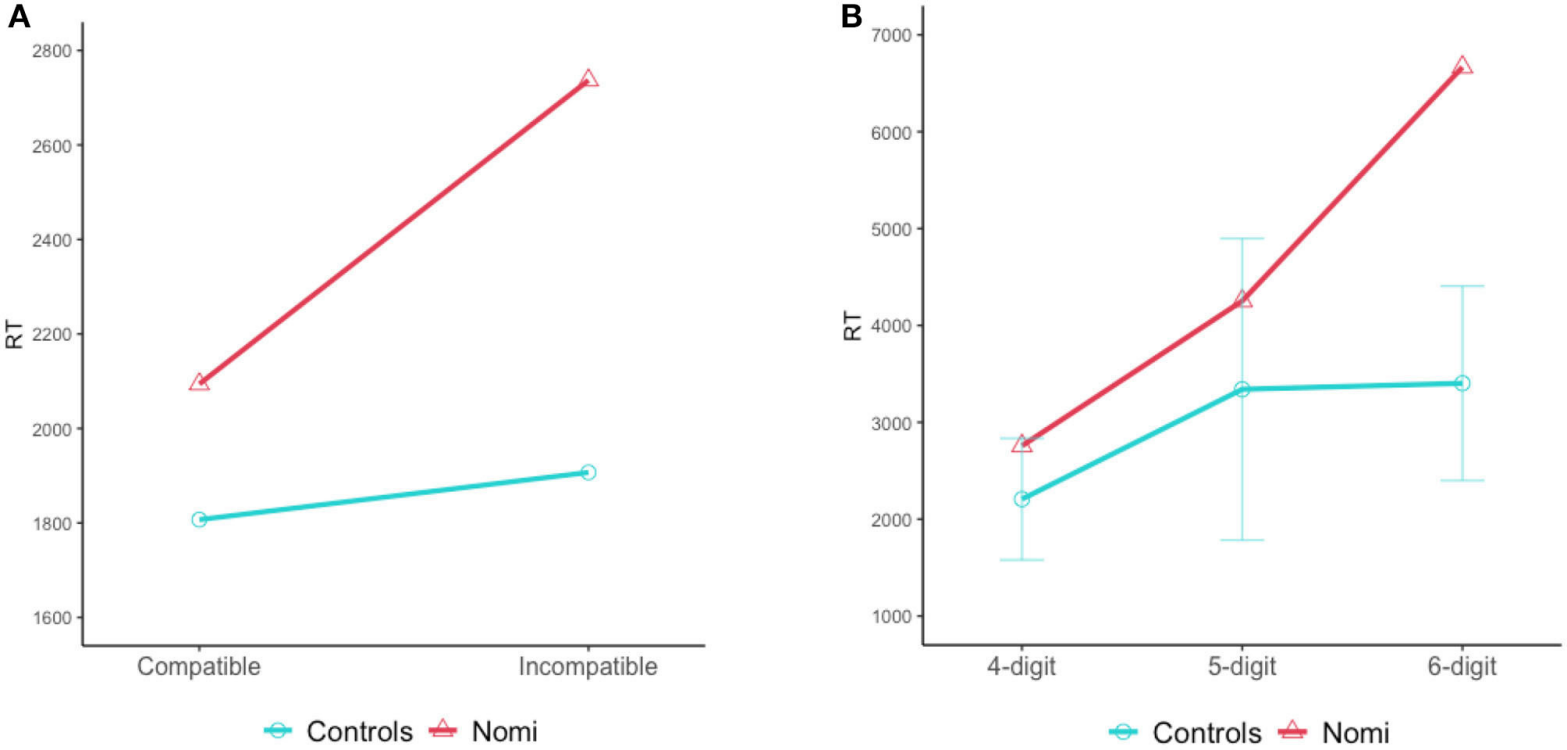
Number-comparison task: Mean RTs of Nomi and the control group. **(A)** RTs in the different-length pairs in the compatible condition (in which the longer number also had a larger first-digit) and in the incompatible condition (in which the longer number had a smaller first-digit). **(B)** RTs in the same-length pairs with similar first-digit, of 4-, 5-, and 6-digit numbers.

Very interesting findings, however, emerged from her pattern of response times in the various conditions. The controls had similar RTs for both types of different-length pairs (the compatible and incompatible conditions), and in fact, 4 of the 10 control participants even had lower average RTs for the incompatible condition, resulting in a relatively small difference between them^2^ (Mean difference = 99 ms, SD = 277). In marked contrast, Nomi had much longer RTs on the incompatible condition, and the difference between her average RTs in the incompatible and compatible conditions was significantly larger than that of the controls (Nomi's mean difference = 643 ms, Crawford and Howell's *t*(9) = 1.87, *p* = 0.047, Figure 6).

This suggests that whereas typical readers can perform number comparisons on the basis of decimal structure, and therefore are less affected by the first digit when there is a difference in decimal structure, Nomi found it more difficult to rely on the decimal structure of the longer numbers (of 5- and 6-digit numbers) and was therefore more affected by the first-digit in these numbers (possibly because she was relying on the identity of the digits, and specifically on that of the leftmost ones). This type of stimuli was exactly the type that was difficult for her in the >5500 task (Section 4.6.1) as well (numbers longer than 5500 but with a first-digit smaller than 5).

In addition, Nomi's RTs were significantly longer than the controls' in the same-length conditions in pairs of 6-digit numbers, which was most prominent with 6-digit numbers that had the same first-digit and differed only in the second- or third-digit (controls: M = 3,403 ms, SD = 1,004 ms; Nomi: M = 6,667 ms, SD = 726, Crawford and Howell's *t*(9) = 3.10, *p* = 0.006). As depicted in Figure 6, her RTs in the same-length 4- and 5- digit numbers (same first-digit) were similar to the controls' and far shorter than her RTs for the 6-digit numbers.

This forms another indication of her difficulty in processing of long numbers of 6-digits, when building the decimal frame is necessary, and yet another indication that impairment of decimal-frame-construction for longer numbers affected not only her reading, but also her comprehension.

### 4.7. The Deficit Is in Creating a Number Frame From Written Numbers: Intact Number Production and Comprehension When the Decimal Positions Are Provided

#### 4.7.1. Reading the Same Multidigit Numbers Presented With a Comma Separator

If Nomi's decimal shifts and decimal structure errors in reading-then-signing originate in a deficit in parsing the number into a number frame, adding a comma separator should help her parse the number into triplets, which would help her in creating the appropriate number frame, so she is expected to make fewer decimal shifts than in reading numbers without a comma (see Dotan and Friedmann, 2019, for similar rationale).

##### Method

Nomi read aloud the same 60 numbers that she had read in the baseline task, but here the numbers were presented with a comma separator between the thousands and hundreds digits (e.g., *12,592*, whereas in the experiment in the baseline task it was *12592*) (multidigit number reading with comma separator A, MAYIM battery, Dotan and Friedmann, 2014). Just like in the baseline task, the numbers were written one above the other, and Nomi was asked to read each number and then sign it in ISL.

##### Results

Nomi made only 2 decimal errors in reading the numbers with a comma separator—one with a 4-digit number, and the other with a 6-digit number—and she immediately self-corrected both of these errors. In contrast to her performance when reading these numbers without a comma separator (in the baseline task), here she was able to read all numbers without giving up and declaring she could not sign them, even in the longest numbers. Her reading of the same 5–6 digit numbers with a comma separator was significantly better than her reading of these numbers without a comma (McNemar test *p* = 0.0001). In addition, Nomi reported that reading the long numbers with a comma separator was much easier for her than reading them without it.

This result supports the conclusion that her deficit was related to the conversion of the number and parsing it into the decimal frame, and it also lends further support for the conclusion that she had no phonological output deficit.

#### 4.7.2. Intact Comprehension of Written Numbers With a Comma Separator: > 5500

##### Method

Nomi was presented with the same multidigit numbers as in the test in the written >5500 test (Section 4.6.2). The numbers were printed scattered on a sheet, however, this time they appeared with a comma separator between the thousands and the hundreds digits. Nomi was asked to mark all numbers that were greater than 5500.

##### Results

Nomi marked correctly all the numbers >5500, including the 5-digit numbers with the first-digit smaller than 5, which she missed in the version written without the comma separator.

These two tests indicate that when an indication as to the decimal structure is given in the written number, reading and comprehension improve considerably and the decimal errors almost disappear. Another result we reported above in section 4.5.1 supports the same point: when Nomi read multidigit numbers presented as Hebrew number-words rather than as written Arabic numerals, she signed them without decimal errors. Notice that the Hebrew number-words provide the abstract decimal structure of the number but not the decimal word-frame of the verbal-signed number in ISL. Nevertheless, once the decimal structure was provided to her (in a separate system- a written spoken language), she did not make decimal errors. A summary of Nomi's performance in all multidigit number tasks is given in Table 1.

### 4.8. Dissociation Between Number Reading and Word Reading

We have established that Nomi has a deficit in the conversion of the written number to its decimal frame. It is interesting to examine whether her deficit is selective to number-reading or whether she also has a deficit in reading words and converting them to their verbal representation.

Dotan and Friedmann (2019) reported double dissociations between dysnumeria in various loci in the number-reading model and dyslexia in reading words in parallel word-reading components. Here we examine whether such a dissociation can also be found between reading-then-signing written numbers and reading-then-signing written words.

#### 4.8.1. Reading and Signing Written Hebrew Words

##### Method

Nomi was asked to read-then-sign a total of 406 written Hebrew words, presented in 5 tests (adapted from the TILTAN battery, Friedmann and Gvion, 2003). All these tests included lists of single words, presented one above the other. Nomi was requested to read each word and sign it in ISL. Below we describe each of the five tests. Nomi's reading performance was compared to control groups of hearing adults (see the number of participants in each control group and their average performance on each test in Table 2); In two of the reading tests, her performance was also compared to a group of 7 deaf native ISL signers (4 women and 3 men, aged 18-44). The five tests were:

1. **Tiltan Siman screening**: 92 words sensitive to different types of dyslexia (including migratable words, irregular words, long words, morphologically-complex words, function words, abstract words, words with many orthographic neighbors, words with double letters and more).
2. **Migratable word list**: 160 migratable words, in which a transposition of two letters creates another existing word (e.g., *stakes*, which can be read with a transposition as “skates,” Friedmann and Gvion, 2001; Friedmann et al., 2010).
3. **Surface list**: 86 potentiophonic and homophonic Hebrew words. Potentiophones are words that if read via the sub-lexical route can potentially be read as other existing Hebrew words, which sound differently (e.g., *none*, which may be read via grapheme-to-phoneme conversion as “known,” Friedmann and Lukov, 2008; homophones are words that sound the same but have different meaning and spelling, so they translate to different signs).
4. “**Artichoke”- long and morphologically complex words**: 36 long and morphologically complex Hebrew words of 5–11 letters.
5. “**Duvshaniyot**”- **morphologically complex words** with derivational morphology: 32 short and long words with Hebrew derivational morphology, in which the same Hebrew root put in a different morphological pattern leads to a change in meaning (e.g., *paired* and *impair*). This type of words should elicit errors in translation to signs if the morphological template is incorrectly identified. If the structural process that creates a multidigit number frame is similar to the process creating the morphological structure of a word, then a dissociation in this task would be especially telling.

**Table 2:** Nomi's performance (percentage correct performance and error types) and the control groups' performance in the Hebrew word reading tasks.

**Test**	**Sensitive to…**	**Total words**	**% correct performance**	**Error types**	**Deaf signer controls M(SD)**	**Hearing controls M(SD)**
**Read-then-sign tasks**						
Screening: single words	Various types of dyslexia	92	98%	1 morphological error, 1 low-frequency Hebrew word that she did not know how to translate into sign	–	*N* = 1,045 98.1% (2.0%)
Migratable words	Letter position dyslexia	160	96%	3 semantic errors (signing a sign semantically related to the target word), 2 letter migrations, 1 omission of a double letter, 1 morphological error	*N* = 7 96% (2%)	*N =* 698 96.6% (2.3%)
Homophone and potentiophone	Surface dyslexia	86	99%	1 letter omission	*N =* 6 97% (3%)	*N =* 257 93.7% (4.1%)
Long words (5–11 letters)	Buffer deficits	36	100%	–	–	*N =* 35 97.6% (2.4%)
Morphologically complex words	Morphological difficulties	32	100%	–	–	*N =* 20 97.5% (1.7%)
**Same-different task**						
Same-different task	Letter position dyslexia, letter identity dyslexia, and word length	40 different pairs	100%	Did not miss any difference between words	–	*N =* 24 97%, 3%

##### Results

Nomi's word-reading was excellent, even compared with hearing Hebrew readers, and very different from her number-reading. Nomi's performance in the five word-reading tasks as well as the analysis of the (few) errors she made in each of the tests are summarized in Table 2. In total, in reading 406 words with various kinds of complexity, Nomi made only 10 errors (98% correct), a rate that is well within the range of typically-hearing control adults. For each of the tasks, her performance was similar to that of hearing controls, and to that of the deaf signers, in both groups her performance puts her in the higher range of performance (*p* > 0.05 for all comparisons between her performance and the control groups using Crawford and Howell, 1998, *t*-test).

Three types of errors in word-reading may be parallel to the errors Nomi made in reading numbers: morphological errors, letter transpositions, and length errors, manifesting in letter omissions or additions. As summarized in Table 2, she did not make any of these errors in a rate higher than the controls:

1. she made only 2 morphological errors in all 406 words she read, and no morphological errors in the Artichoke and the Duvshaniyot tests, which were created to examine the reading of morphologically complex words. This indicates that she does not have a general problem with reading structurally complex items, but rather a deficit that is limited to the structure of (long) multidigit numbers.
2. In the migratable words test, which was created to elicit letter migrations within words, as all the 160 words in the test were “migratable,” she made only 2 letter migrations, again, better than the average performance of hearing readers.
3. In the long-word test she made no reading errors at all, and she did not make more letter omissions or additions than the typically hearing controls, indicating that her length perception of words was unimpaired too.
  So not only the general percentage correct performance but also the analysis of her error types indicates a clear difference between her word reading and her number-reading.

Additionally, in the Artichoke and the Duvshaniyot tests, Nomi made no errors at all, not even in the longest words. Comparing her reading of 5–6 letter words to 5–6-digit numbers (a total of 5 errors out of 190 words, compared to 26 errors out of 54 numbers), her word reading was significantly better, χ^2^(1) = 74.50, *p* << 0.0001) when taking into account all words of 5 letters or longer yielding a total of 6 errors out of 217 5–11 letter words, this difference is even greater, χ^2^(1) = 81.22, *p* << 0.0001). This indicates a clear dissociation between her reading of long multidigit numbers and her reading of long words, and shows that these two processes are separate.

#### 4.8.2. Same-Different Decision: Pairs of Written Words Presented Together

##### Method

To assess Nomi's orthographic-visual analyzer and to further compare her word- and number-reading, we administered a same-different task with pairs of written words. Nomi was presented with 60 pairs of Hebrew words printed one next to the other in a list, and was asked to mark the pairs in which the two words were identical. The test included 10 words that differed in length (length difference created by doubling a letter in one word that created the other word, similar to driver-diver or diner-dinner), 10 that differed in one letter identity, 20 that differed in the order of two adjacent letters (e.g., flies-files, skates-stakes), and 20 identical pairs.

##### Results

Nomi did not miss any difference between words (she did not mark any pairs that were not identical), and she only missed 2 pairs of identical words (3% of the total number of words, which falls well within the results of hearing controls (*n* = 24, mean errors = 3%, SD = 3%, Lorber, 2020). This supports that she has very good reading, and she does not make errors of letter-identity, migrations of letters within the words, or omissions of double letters which would indicate number length deficit.

#### 4.8.3. Interim Summary: Words vs. Numbers

To summarize, Nomi's word-reading was very good. It was similar to that of the higher range of hearing controls and of deaf native signer controls. Her general performance in the word-reading tasks, then, points to a clear dissociation between her good word-reading and her poor number-reading. We also found that the component that was impaired in her number-reading did not cause a parallel impairment in word-reading: if processes related to the decimal structure of numbers are parallel to word structure –morphology, she did not make morphological errors in reading words, indicating that the structural component is not shared by numbers and words.

Deficits in the conversion of number-length could be envisaged also as parallel to deficits in word-length, which should have led to errors of letter addition or omission, which may be similar to number-length errors, and errors of letter-position which may be similar to decimal shifts in reading numbers. Here, too, Nomi did not make such errors more than the controls.

Therefore, in each of these subprocesses she showed intact performance in word-reading: she made no morphological errors in the two tests with morphologically complex words, she made only two letter omissions of the 406 words she read, she made fewer letter transpositions than the hearing control average in the migratable word test, and she did not miss any difference in letter-order or in word-length in the same-different task, indicating that there was no shared component that was impaired, and that her impairment was selective to number-reading.

## 5. Discussion

We brought here the first report of a specific type of dysnumeria—an impairment in number-processing, in a deaf signer, Nomi. Nomi's dysnumeria manifested itself in a difficulty to read and comprehend the decimal structure of long multidigit Arabic numbers.

Her most prominent error type in multidigit number reading was decimal errors—she had difficulty in processing the decimal structure of the number, most notably in longer numbers of 5–6 digits.

### 5.1. Nomi's Functional Locus of Deficit in the Number Reading Model

**Figure 7:**
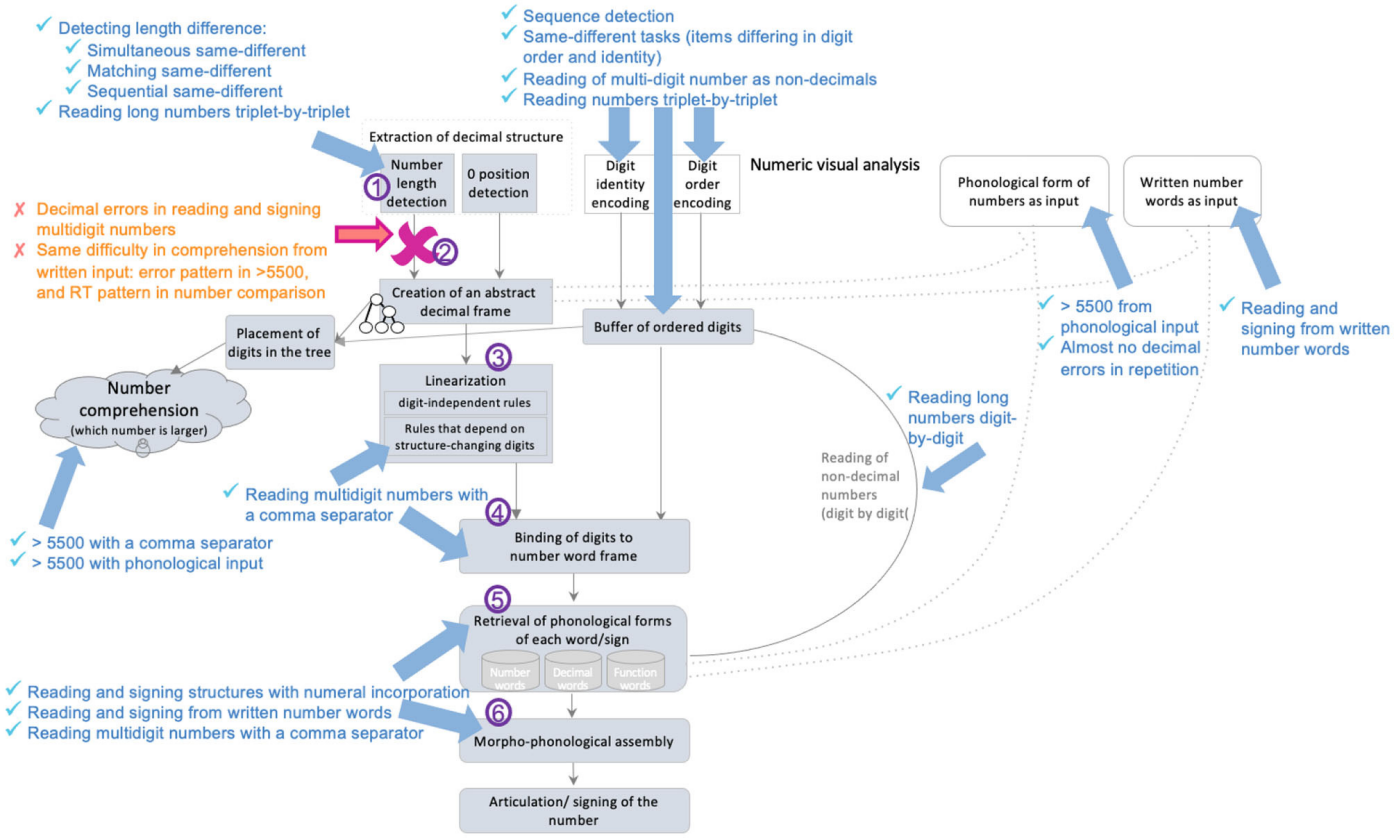
The number reading model with Nomi's performance related to each of the components. In blue: performance that is within the control range; orange: impaired performance, lower than the control group. Circled numbers indicate theoretically possible sources of decimal errors in multidigit number reading. Crimson X indicates the locus of Nomi's deficit in view of her performance in the various tasks.

We suggest that Nomi's impairment lies in the conversion of written multidigit numbers into the abstract decimal frame, namely in the connection between the (intact) numeric-visual analysis stage of extraction of decimal structure and the decimal structure component. This deficit is marked (2) in Figure 7, which already uses a modified model that will be motivated and explained below. As we discuss below, we assume that the construction of the non-verbal decimal frame of the number serves both reading aloud and comprehension of written numbers, and therefore a deficit in the connection from visual input to this process affects both reading aloud and comprehension from written input.

Nomi showed difficulties in reading multidigit numbers, mainly in longer numbers of 5–6 digits. Her errors were mostly decimal shifts of the leftmost digit (e.g., reading Arabic numbers such as 34567 as three thousand…), resulting in saying the first digits in an incorrect (and smaller) decimal position.

As depicted in Figure 7, decimal shift errors could, theoretically, result from three general stages in the number-reading process: the numeric-visual input processes of extracting number-length ①, the conversion of a written number to its abstract decimal frame ②, or the verbal-output processes ③–⑥: in linearization of the abstract frame into a verbal number frame ③, in misalignment of the ordered digits into the number frame during the binding process ④, in the retrieval of number-words/signs (retrieving/producing a word with incorrect class) ⑤, or in holding too many number-words/signs at the same time ⑥.

We will now use the results of the tests reported above to show how we reached the conclusion that Nomi's deficit is in the conversion of written numbers to the decimal frame and why the other theoretically possible loci are excluded for her deficit. Figure 7 summarizes all the test results upon which we base our conclusions about spared and impaired components, which we describe and discuss in detail below (blue for good performance, orange and crimson for impaired performance).

The early numeric-visual analysis stage is ruled out as the source of Nomi's deficit on the basis of her good performance in tasks that involve the numeric-visual analysis without the later conversion and phonological output stages. She performed very well in the three same-different tasks, and specifically in detecting pairs that differed in number-length or in digit-order, suggesting she did not have deficits in extracting number-length and digit-order from written numbers. She also performed well in digit-sequence decision. Her good performance in reading the same numbers when signing them non-decimally, digit-by-digit or triplet-by-triplet, provides further strong evidence that her numeric-visual analysis was intact. Her deficit emerged only when she had to use this information to read the number “aloud” as a multi-digit number, namely, when she had to create the decimal number frame for the written number (for production, and, as we will show below, also for comprehension). A deficit in the numerical input buffer is also excluded in view of the absence of order- and identity-errors in all input tasks.

The verbal output processes are also ruled out as the source of Nomi's decimal errors: when she produced long multidigit numbers in tasks that did not involve written Arabic numerals (in reading numbers written as Hebrew number words and in repeating multidigit numbers), she made no decimal errors. She could sign without any decimal errors long multidigit numbers written in Hebrew words (of the same length as she failed to sign in reading). Multidigit number repetition was not easy for her, but still, she made almost no decimal errors in two tasks of multidigit number repetition. These results demonstrate that Nomi can produce multidigit numbers with their correct decimal structure when no Arabic numeral reading is involved.

Several additional findings support the conclusion that her production is intact. When she read the same multidigit numbers with a comma separator, she made significantly fewer decimal errors. Reading numbers with comma requires the same output processes as reading numbers without comma, so her difficulty in numbers without comma could not have resulted from a deficit in the output processes.

Another indication of her intact phonological output buffer is her good reading-then-signing of structures involving number incorporation (like “twice”), as number incorporation takes place in the phonological output buffer (Haluts and Friedmann, 2020).

Her good production of number incorporation, which are morphologically-complex structures involving numbers, also demonstrates that her decimal errors did not stem from a deficit in the processing of morphologically complex structures that involve numbers. This conclusion is also supported by her good production of the morphologically complex decimal numbers in repetition and reading-then-signing of Hebrew number words.

Finally, in reading multidigit numbers with fewer number-words (e.g., 400300), she still made many decimal errors, in a rate similar to the error rate she had in reading multidigit numbers with many different digits. These numbers include the same number of written digits but require far fewer number-signs in production, so these numbers should have made a difference only for the phonological output stages, and should have been easier had the deficit been in the phonological output stages. The finding that Nomi still made many decimal errors in reading these numbers suggests that the source of her decimal errors was not the need to hold and assemble many number-signs together.

The pattern of Nomi's errors can be taken as additional evidence that Nomi's decimal errors did not result from a phonological output deficit: had her deficit been in the retrieval of number-signs in the correct class, we would not expect errors only on 5–6-digit numbers, and we would not expect errors mainly in the leftmost digit, and toward a smaller decimal position. Additionally, had she had a deficit in lexical retrieval, we would expect other phonological substitutions, such as other number-identity errors (signing THREE instead of FIVE), which almost never occurred.

We therefore conclude that Nomi's deficit in number-reading (reading-then-signing) lies in the creation of the decimal number frame from written number input. That is, in the conversion of the decimal information extracted in the (intact) numeric-visual analyzer into a number frame. The findings that: (a) Nomi made errors only in numbers of 5-digits and up, and had almost no decimal errors in 4-digit numbers and no errors in 3-digit numbers, and (b) her decimal errors were always in the direction of using a number frame that is smaller than the written number (in one decimal position) suggest that her deficit was a result of a limitation in the size of the number frame into which she could place the written numbers. She is able to build a smaller tree/number frame from written input, so the frame for shorter numbers (of 4 digit or less) is created correctly. However, for 5- and 6-digit numbers she cannot create a frame that would be suitable for the written input. She typically created a 4-digit frame for the 5-digit numbers, and never succeeded to create decimal structures for 6-digit numbers, mainly saying “I can't,” starting and failing to create a 5-digit tree, or breaking the number into two triplets. This treelet deficit might be similar to Power and Dal Martello (1997) study that showed, for 7-year-olds, decimal errors that result from their inability to create larger trees. Nomi's impairment pattern is possibly similar also to that of ED (Dotan and Friedmann, 2018, 2019), who also had decimal errors, and mainly on the longer numbers, but ED made 26% errors in reading 4-digit numbers, so she might have had difficulty with even smaller decimal frames compared to Nomi.

This deficit can be conceptualized either as a deficit in the conversion from the numeric-visual analysis stage to the number frame, or as a deficit in a component of number frame building for written numbers^3^. At this point we do not see a way to distinguish between decimal-frame construction that is input-specific (written numbers, phonologically presented numbers), and a single decimal-frame construction component that has separate connections from the different inputs.

### 5.2. Nomi's Multidigit Number Comprehension and Its Implication for the Model

Multidigit number comprehension, as measured by number-comparison tasks, was impaired along the same lines as Nomi's reading “aloud”: just like her decimal errors in reading 5–6-digit numbers, she also had difficulty in comprehending the decimal structure of numbers of this length. Whereas her comprehension of short, 3–4-digit numbers was intact, she failed to detect that 5-digit numbers were larger than 4-digit numbers when their first digit was not larger than the first digit of the 4-digit numbers (23675>5500) in two different tasks. This difficulty manifested itself both in errors (in the >5500 task) and in response time patterns. This suggests that her deficit in processing the decimal structure of the written numbers also affected her comprehension of number-size.

What is the source of this deficit in comprehension? The data showed that her early numeric-visual stage of number-length encoding (as well as digit-identity and order) was intact. So just as the deficit in reading “aloud” could not have emerged from the numeric-visual stage, neither could the comprehension deficit. Her comprehension of the same numbers from signing was also intact, indicating that the deficit was not in the comprehension itself but was limited to written numbers.

It therefore seems that the source of the deficit in number comprehension was impaired conversion of the written number into its decimal frame, just like in number-reading. This suggests the possibility that the deficits in reading and in comprehension are in fact a single deficit, at a component that is shared by the two processes. Conversely, it might be that there are two independent identical impairments of converting the numeric-visual information of multidigit numbers into their decimal form—one in conversion for reading, the other in conversion for comprehension, and both manifest themselves in numbers of the same lengths and in similar errors. Whereas this two-deficit option is possible, Ockham's razor drives us to prefer the option of a deficit in a component shared by reading and comprehension. We therefore suggest that Nomi's deficit lies in the conversion of information from the numeric-visual stage into the shared decimal number frame construction stage, thus affecting both reading “aloud” and comprehension.

### 5.3. Implications for the Number Reading Model

**Figure 8:**
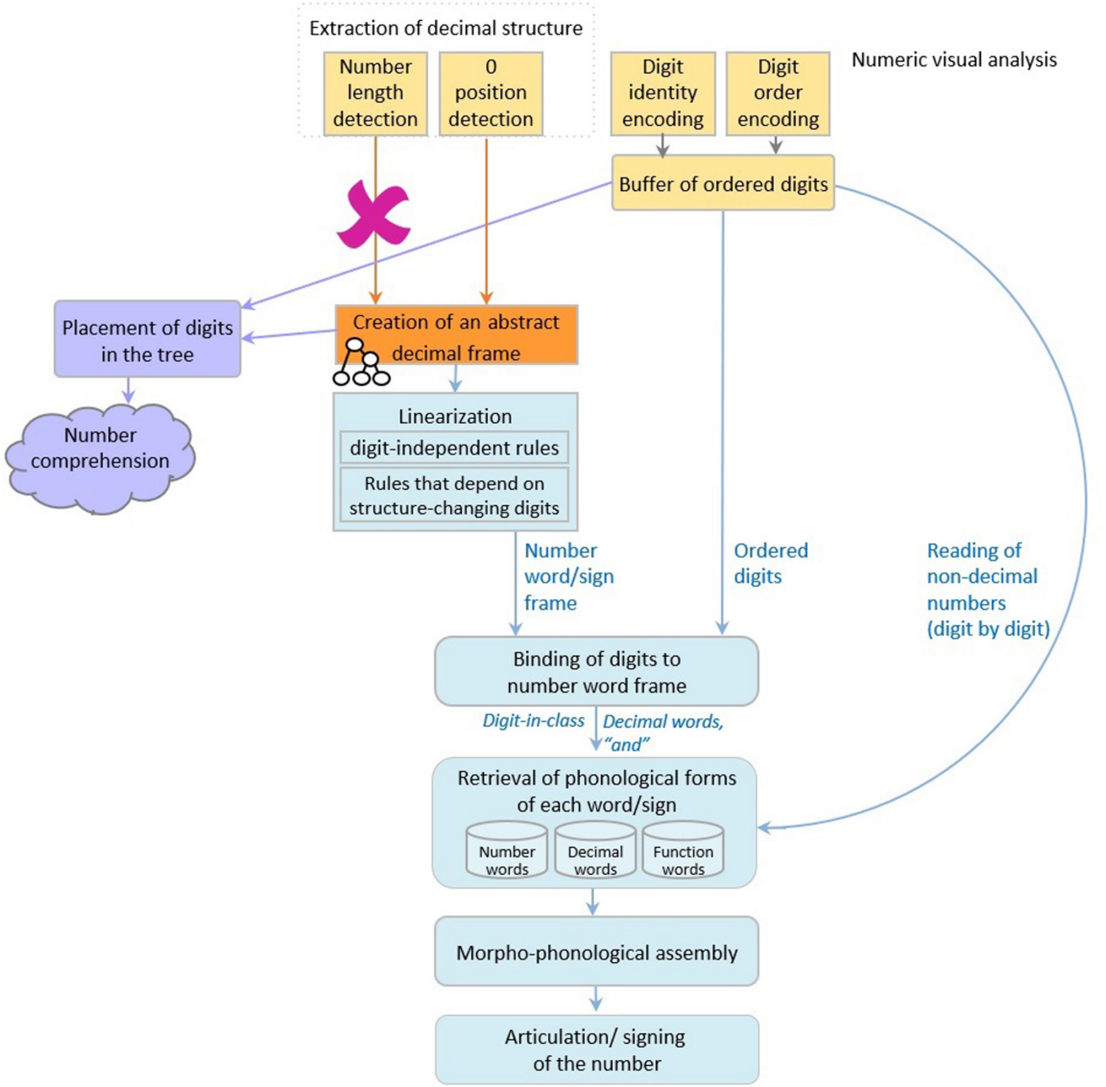
A revised number reading model. Three modifications to Dotan and Friedmann's (2018) model: (1) The decimal structure building component is non-verbal, and it is shared with comprehension processes, (2) an addition of a non-decimal number reading route, (3) triplet parsing is no longer part of the numeric-visual analysis stage.

Nomi's pattern of impairment thus offers new insights to the number-reading model. The first insight relates to the number-frame-building component shared by reading aloud and comprehension, described in the previous section. In Figure 8 we provide a possibility for the architecture of such shared component: the creation of the number frame is abstract and non-verbal and is shared by reading and comprehension. This abstract, non-verbal, number frame creation component provides the information of the decimal structure of the number both for later verbal stages (in signs or spoken-words) and for comprehension (in our case, number comparison), so it is connected to the linearization component, which is verbal and language-dependent, and to the further comprehension components.

The idea of a ***shared abstract decimal-structure component***for comprehension and production is in line with McCloskey's (1985, 1986, 1992) idea that reading aloud of multidigit numbers passes through an abstract stage that is shared with comprehension. Differently from McCloskey, and in line with Cohen and Dehaene (1991) and Dehaene and Cohen (1995), we do not assume that this shared component is a semantic representation that follows, and requires, “number comprehension,” but rather a stage that immediately follows numeric-visual analysis, preceding both production and comprehension of written multidigit numbers. We suggest that this stage involves the construction of an abstract, non-verbal, decimal number frame.

Once we assume such an abstract number frame component, we can assume it is responsible both for the decimal structure and for the parsing into triplets. We currently do not see the need to assume a separate triplet-parsing component at the numeric-visual analysis stage, and patients like ED (Dotan and Friedmann, 2018, 2019), who showed impaired triplet-parsing, may be impaired in the general process converting the information from the numeric-visual analysis onto the number frame (at the moment we remain agnostic as to whether there is a separate triplet-parsing component, whether it resides in the numeric-visual input or in the phonological output components or both, and whether it depends on the way the target language divides numbers into groups).

A second insight from this study is the existence of ***a non-decimal route***, which allows a digit-by-digit reading of multidigit numbers, without forming a decimal representation (somewhat similar to the non-lexical route in word-reading, Coltheart et al., 2001). Nomi had a deficit in reading multidigit numbers with decimal structure, but had no problem reading the same numbers digit-by-digit. A similar pattern was reported for the patients in Cohen and Dehaene (1995), but for digit-identity errors rather than decimal errors. We suggest that this digit-by-digit reading is performed in the non-decimal route, portrayed in Figure 8 with an arc connecting the numeric buffer of ordered digits with the phonological output component, which bypasses the decimal structure construction.

### 5.4. Dissociation With Reading Words

In marked contrast to her impaired multidigit number reading, Nomi's word reading was intact. Her performance in reading 406 words did not differ from that of typically-hearing Hebrew speaking adults, nor did it differ from the reading of deaf signers. This already shows a clear dissociation between her poor multidigit number reading and her very good word-reading.

Additionally, when one examines the pattern of errors that would have been expected had she had a deficit in word reading that is parallel to her deficit in number-reading, it is clear that she does not make similar error types: she made only two letter omissions, two letter transpositions, and two morphological errors (1.5% together) which could be counted as parallel to decimal errors in numbers, compared to 20 out of 100 (20%) decimal errors in her number-reading.

Additionally, her deficit in number-reading was most pronounced in the longer, 5–6-digit numbers. Conversely, her word reading of 5- and 6-letter words (and even longer words) was unimpaired and significantly better than her reading of numbers of similar lengths.

These results, thus, support the conclusion that number-reading is implemented, at least in part, by mechanisms that are different and separate from the ones that are used in word-reading (Friedmann et al., 2010; Shum et al., 2013; Abboud et al., 2015; Hannagan et al., 2015; Güven and Friedmann, 2019; for a review, see Dotan and Friedmann, 2019).

### 5.5. A Specific Impairment in Number Reading in a Deaf Signer

Nomi, a deaf user of a sign language, showed a specific impairment in number-reading. As discussed above, Nomi's impairment resulted from a very specific stage in the number-reading model. Her impairment seems similar to that of NR, reported by Noel and Seron (1993) and discussed above in the introduction, and to that of ED, reported by Dotan and Friedmann (2018, 2019). The fact that very similar number-reading impairments can be found in spoken language users and in sign language users suggests that the mechanisms that process number reading are shared by all speakers of human languages and do not depend on the modality in which the language is transmitted.

### 5.6. Conclusion

We reported here of the first in-depth investigation of a selective dysnumeria in a user of a sign language. Her pattern of errors and performance in various tasks indicated a decimal-structure conversion dysnumeria, a deficit in the construction of decimal number frame from written numbers of 5-digits or longer. Her deficit was shared by reading aloud (reading-then-signing) and comprehension processes. Nomi made no errors in tasks that did not require the construction of decimal frames from written numbers: she performed well in tasks involving only the numeric-visual analysis, and made virtually no decimal errors in tasks involving the production of multidigit numbers without written input. When she read the same long multidigit numbers with cues as to the decimal structure, she made fewer decimal errors, and when she read the exact same numbers in a non-decimal system in ISL, which only involves digit-by-digit signing, she made no such errors. These results indicate that prior to the construction of the verbal number frame, a non-verbal abstract frame is constructed, which is shared by reading and comprehension. Additionally, these results provide evidence for a parallel, non-decimal reading route for reading multidigit numbers. The assessment of dysnumeria in sign language, thus, opened a new window to insights regarding the number-reading process.

## Data Availability Statement

The datasets presented in this article are not readily available due to privacy issues. Requests to access the datasets should be directed to the corresponding author.

## Ethics Statement

The studies involving human participants were reviewed and approved by Tel Aviv University Ethics committee. The participants provided their written informed consent to participate in this study. Written informed consent was obtained from the individual(s) for the publication of any potentially identifiable images or data included in this article.

## Author Contributions

NF, NH, and DL worked together on the conceptualization, creation of tests, testing Nomi, and analyzing the results. NF and NH worked together on all stages of writing. NF provided resources and funding. All authors contributed to the article and approved the submitted version.

## Conflict of Interest

The authors declare that the research was conducted in the absence of any commercial or financial relationships that could be construed as a potential conflict of interest.

## Footnotes

^1^Zero-position errors, e.g., 8030 → 8003, which occurred 4 times in the control's reading (and never in Nomi's reading) were coded separately from decimal shifts.

^2^This compatibility effect may be in line with other compatibility effects of irrelevant digits (e.g., Nuerk and Willmes, 2005), and with other effects of irrelevant properties on RTs (e.g., Domahs et al., 2010, 2012) in size judgment tasks.

^3^It is also possible to conceptualize the locus of this deficit as a deficit in number length analysis of the numeric-visual analysis system in a process that **only** applies to reading aloud and comprehension of multidigit numbers with their decimal structure (but not to same-different decision tasks). We find such conceptualization inferior to a deficit in the connection between the analyzer and the decimal frame building, because it requires assuming that the number length analysis stage functions differently in different tasks and “knows” already what the task is and whether it requires multidigit numbers as phonological output (or size comprehension) or not. It also requires that the early length detection stage in the numeric-visual analysis includes a function that serves late stages such as reading aloud and comprehension but not same-different decision. Assuming that the deficit resides in the connection of the length detection component to the frame building component does not require such less-probable assumptions.

## A. Appendix

**Table A1:** Variants of digit-signs in ISL.

	**Variant 1**	**Variant 2**	**Variant 3**
**0**			
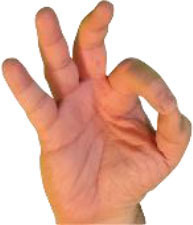
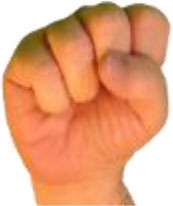
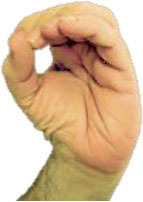
**1**			
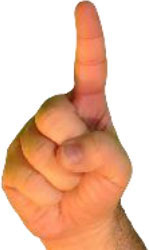
**2**			
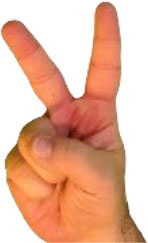
**3**			
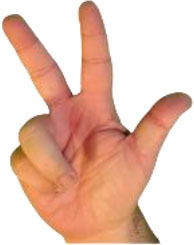
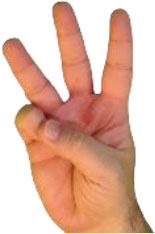
**4**			
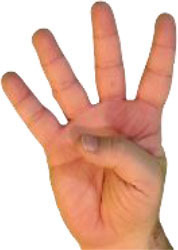
**5**			
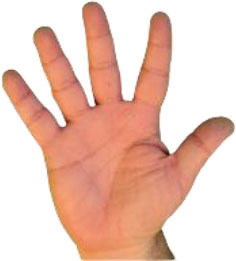
**6**			
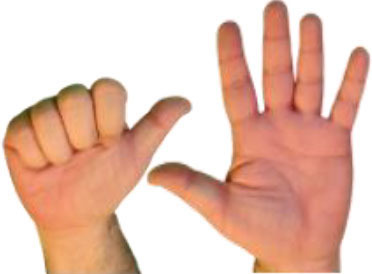
**7**			
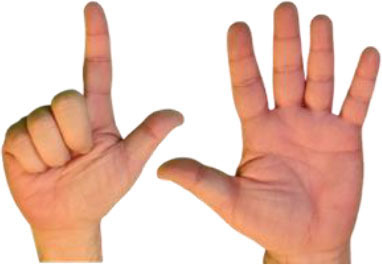
**8**			
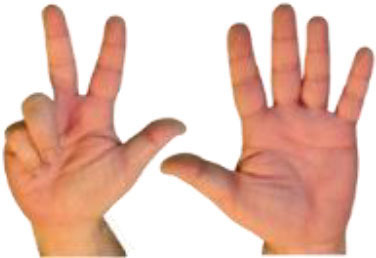
**9**			
